# Microbiome variations induced by delta9-tetrahydrocannabinol predict weight reduction in obese mice

**DOI:** 10.3389/frmbi.2024.1412468

**Published:** 2024-07-16

**Authors:** Avi Kaye, Matthew Rusling, Amey Dhopeshwarkar, Parhesh Kumar, Lauren Wagment-Points, Kenneth Mackie, Li-Lian Yuan

**Affiliations:** ^1^ Department of Physiology and Pharmacology, College of Osteopathic Medicine, Des Moines University, Des Moines, IA, United States; ^2^ Gill Center & Department of Psychological and Brain Sciences, Indiana University, Bloomington, IN, United States

**Keywords:** tetrahydrocannabinol, microbiome, weight loss, sex-specific responses, linear mixed-effects model

## Abstract

**Introduction:**

Obesity and high-fat diets induce consistent alterations in gut microbiota composition. Observations from epidemiological reviews and experiments also illustrate weight regulation effects of delta9-tetrahydrocannabinol (THC) with microbiome shifts. Therefore, we investigated the weight-loss potential of THC in obese mice models and to elucidate the contribution of specific gut microbiome changes in THC-induced weight loss.

**Methods:**

High-fat diet induced obese mice were treated with oral THC supplementation for two weeks and compared with controls. In addition to measuring weight, fecal samples were obtained at various timepoints, sequenced for bacterial 16s rRNA content and analyzed using QIIME2. Alpha and beta diversity were computed followed by linear mixed effects (LME) modeling of bacterial relative abundance relationship to THC treatment and weight change.

**Results:**

In both male and female mice, the THC group had significantly greater average weight loss than controls (−17.8% vs. −0.22%, p<0.001 and −13.8% vs. +2.9%, p<0.001 respectively). Male mice had 8 bacterial taxonomic features that were both significantly different in relative abundance change over time with THC and correlated with weight change. An LME model using three bacterial features explained 76% of the variance in weight change with 24% of variation explained by fixed effects of feature relative abundance alone. The model also accurately predicted weight change in a second male mouse cohort (R=0.64, R^2^=0.41, p=<0.001). Female mice had fewer significant predictive features and were difficult to model, but the male-produced 3-feature model still accurately predicted weight change in the females (R=0.66, R^2^=0.44, p<0.001).

**Conclusion:**

Using a stepwise feature selection approach, our results indicate that sex-specific gut microbiome composition changes play some role in THC-induced weight loss. Additionally, we illustrated the concept of microbiome feature-based modeling to predict weight changes.

## 1 Introduction

With a tripling in prevalence between 1975 and 2016, obesity was declared a global epidemic by the World Health Organization (World Health Organization, 2021). Obesity particularly affects the United States population which suffers from a 41.9% obesity rate as of March 2020 (Centers for Disease Control, 2021). Obesity increases the risk of numerous diseases such as heart disease, type 2 diabetes, stroke and various types of cancer (Mitchell et al., 2011). Even when controlling for diet, variations in the human genome alone are insufficient to explain differences in obesity rates (Gilbert et al., 2018). However, interactions between host genetics and environmental exposures such as diet may strongly influence obesity (Benson et al., 2010). Furthermore, it is evident that immune system function and metabolism are inextricably connected as energy-rich conditions activate adipocytes to release inflammatory mediators like TNF (Hotamisligil and Erbay, 2008). These observations connecting immune function with metabolism have inspired investigations into the influence of gut microbiota on metabolism and body mass.

The gut microbiome is inexorably tied to weight and metabolism regulation (Carmody and Bisanz, 2023). For example, one group of researchers could classify lean versus obese individuals with over 90% accuracy using just host microbial community composition (Knights et al., 2011). Obesity consistently alters the gut microbiome with implications for energy utilization and storage in mammals (Davis, 2016) including humans (Liu et al., 2017; Bisanz et al., 2019). A simple transfer of microbiota from obese mice into lean germ-free mice can induce increased total body fat and energy harvest capacity (Turnbaugh et al., 2006). Conversely, there are patterns of microbiome composition associated with weight reduction and lower levels of inflammation (Ferrarese et al., 2018). Probiotics in obese individuals has been shown to change intestinal microbiota with accompanying energy metabolism remodeling, regulated parasympathetic activity, and increased expression of genes involved in thermogenesis, glucose and lipid metabolism (Sivamaruthi et al., 2019). Weight loss tends to be associated with abundance of a subset of bacterial taxonomies rather than a global composition change (Ferrarese et al., 2018; Stanislawski et al., 2021; Bliesner et al., 2022). Some studies detected increases in alpha diversity – measures of species richness in a population – with weight loss (Koutoukidis et al., 2022) while others detected no difference (Michael et al., 2020).

Various mechanisms have been proposed to explain the connection between gut microbiome composition and weight, highlighting a bidirectional gut–brain axis whose importance has become increasingly clear (Mayer et al., 2022). Centrally, the GI system collects information about the environment and transmit signals via the Vagus nerve to stimulate secretion of peptides like GLP-1, peptide YY and ghrelin that influence feeding behavior (Bonaz et al., 2018; Yin et al., 2022). Central nervous system (CNS) activity in-turn affects intestinal stability including local transport, secretion and permeability via autonomic, endocrine and immune pathways that influence microbiome composition (Bonaz et al., 2018; Yin et al., 2022). In the gut, intestinal microbiome regulates local physiology through interactions with the Enteric Nervous System (ENS) interactions (De Vadder et al., 2018). Numerous bacteria can break down carbohydrates, especially in the phyla Bacteroidetes which is decreased with malnutrition (Ottman et al., 2012). Specific bacteria can produce short chain fatty acids (SCFA) such as butyrate which increases mitochondrial activity, improves insulin sensitivity, prevents inflammation, induces gut motility and increases intestinal barrier function (Registad and Kashyap, 2013; Chakraborti, 2015). SCFAs also impact ENS integrity by promoting neuronal survival and neurogenesis (Vicentini et al., 2021). Amino acid metabolism is also involved in weight regulation as evidenced by decreases in glutamate-processing bacteria in obese mice and humans (Liu et al., 2017). Additionally, lipopolysaccharides (LPS) released from the cell wall of gram-negative bacteria modulate intestinal function and signaling (Brown et al., 2023). The endocannabinoid system (ECS) plays an integral role in the gut-brain axis, impacting weight regulation and microbiome composition (de Azua and Lutz, 2019).

The ECS is a regulatory network of lipid mediators that primarily act as retrograde messengers to regulate pre-synaptic neurotransmitter release (Lu and Mackie, 2021). The two main cannabinoid receptors, CB1 and CB2, are inhibitory G proteins with CB1 primarily expressed in the CNS and CB2 on immune cells (Vemuri et al., 2008). The best characterized endogenous endocannabinoids (eCBs) include 2-arachidonoyl glycerol (2-AG) and N-arachidonoyl ethanolamine (AEA). However, much of our knowledge about the ECS is derived from research on delta9-tetrahydrocannabinol (THC), the main psychotropic component of cannabis. The affinity and efficacy of THC varies from that of eCBs, causing each to have a unique pharmacology (Lu and Mackie, 2021). The ECS broadly influences mammalian physiology (Mir et al., 2023). For instance, eCB receptors and ligands are present in the ENS, gut epithelium and enteroendocrine cells such as glucose-dependent insulinotropic polypeptide cells (K cells) and cholecystokinin (I cells). These, in turn, modulate brain functions indirectly via circulating metabolic factors and directly via activation of the Vagus nerve (Cuddihey et al., 2022; Srivastava et al., 2022). Accordingly, the ECS strongly regulates gut motility and permeability, energy homeostasis through glucose and lipid metabolism, and inflammation (Cuddihey et al., 2022; Srivastava et al., 2022; Mir et al., 2023). eCB activity can thereby modulate metabolism and body weight by diverse mechanisms (Cani et al., 2016; Iannotti and Di Marzo, 2021). Furthermore, there is strong evidence that microbiota composition influences activity of the eCB system and vice versa (Hasenoehrl et al., 2016).

The ECS is dysregulated in metabolic syndrome and obesity with increased total levels of circulating eCB ligands and inflammatory mediators (Di Marzo, 2008; Izzo et al., 2009; Martins et al., 2015). Mechanistically, increased dietary fat exposure appears to trigger eCB synthesis through Vagus-mediated effects (DiPatrizio et al., 2015). Observations of increased eCB levels in obesity resulted in the development of a CB1 antagonist Rimonabant, that while successfully inducing weight loss, it was discontinued due to the adverse psychiatric effects (Foll et al., 2009). Interestingly, despite THC’s reputation as an appetite stimulator, epidemiological studies reliably demonstrate a decreased body mass index (BMI) in chronic cannabis users compared with non-users (LeStrat and Le Foll, 2011; Sansone and Sansone, 2014; Alshaarawy and Anthony, 2019). A proposed mechanism for reduced BMI with cannabis use is a downregulation of CB1 following chronic THC exposure, thus decreasing sensitivity to the orexigenic actions of eCBs (Clark et al., 2018). Conversely, increased dietary fat exposure and increased incretin levels in obesity upregulate CB1, decrease eCB sensitivity and result in heightened AEA and 2-AG production (Chia et al., 2017). Effects of THC on body mass appear to be dependent on baseline conditions, as low weight individuals with cancer or HIV see an increase in appetite (Sansone and Sansone, 2014) and mice models of anorexia experience less weight loss (Verty et al., 2011). ECS alterations with adiposity appear to be connected with changes in gut microbiome composition due to shared roles in metabolic and inflammatory regulation (Geurts et al., 2011).

Recent studies have elucidated a bidirectional relationship between gut microbiota composition and eCB tone that subsequently influences weight (Ellermann et al., 2020). In general, microbiome dysbiosis induced by antibiotics alters circulating eCB levels while probiotic treatment re-establishes equilibrium (Guida et al., 2018; Manca et al., 2020). On the flip side, alterations in the ECS are known to induce changes in the host microbiome (Dione et al., 2020). The ECS can alter epithelial barrier function and chloride secretion through CB1 receptors in intestinal epithelial cells and can decrease cytokine release via CB2 interactions on immune cells all of which impacts microbe composition (Cuddihey et al., 2022). In regards to obesity, there is strong evidence of a microbiota-to-adipose tissue regulatory loop impacting endocannabinoid system tone. (Muccioli) While the ECS influences gut permeability and adipogenesis, LPS modulates eCB-driven adipose tissue metabolism (Muccioli et al., 2010). Several gut bacterial families such as *Peptostreptococcacaeae*, *Akkermansiaceae* and *Veillonellaceae* are associated with variations in circulating endocannabinoid levels beyond variations in body fat mass and dietary fatty acid intake (Castonguay-Paradis et al., 2020). Additionally, in lean mice, an abundance of bacteria producing SCFA produce anti-inflammatory effects, partially through ECS modulation (Vijay et al., 2021).

Previous experiments demonstrated that THC administration prevented further weight and fat mass gain and microbiome changes in diet-induced obesity (DIO) model mice without impacting gut microbiota or weight in lean mice (Cluny et al., 2015). The purpose of the current research is to investigate the potential for use of THC for weight reduction in obese mice models and to further elucidate the roles of gut microbiome changes in THC-induced weight loss. Current evidence discussed above suggests that THC administration alone will increase weight loss in obese mice accompanied by shifts in microbiota accounting for a significant portion of the change in body mass. A secondary goal of this research is to examine the potential of using advanced statistical modeling of gut microbiome patterns to predict specific functional outcomes – in this circumstance, weight change.

## 2 Materials and methods

### 2.1 Experimental animals and diet

Adult male or female mice (initially purchased from KOMP, UC Davis, Davis, CA and Jackson Laboratory, Bar Harbor, ME, respectively, and bred at Indiana University) on a C57BL/6J background were used in this study. Mice were housed in polycarbonate cages on a 12-hour light:dark cycle and were provided ad libitum access to water and defined high fat rodent chow (containing sucrose and 58% of the total calories from fat) (D12331;Research Diets, New Brunswick, NJ). High fat diet (HFD) began on postnatal day 21. Mice were weighed every week at baseline to assess weight gain. Mice with weight greater than or equal to 40 grams (for females) and greater than or equal to 45 grams (for males) were considered obese and used for experiments. At the age between 90 and 120 days, these mice on HFD reached the weight threshold mentioned above. Animals were maintained on HFD throughout the experimental study unless otherwise stated. All experimental procedures were approved by the Institutional Animal Care and Use Committee of Indiana University Bloomington.

### 2.2 Oral THC treatment

**Figure 1:**
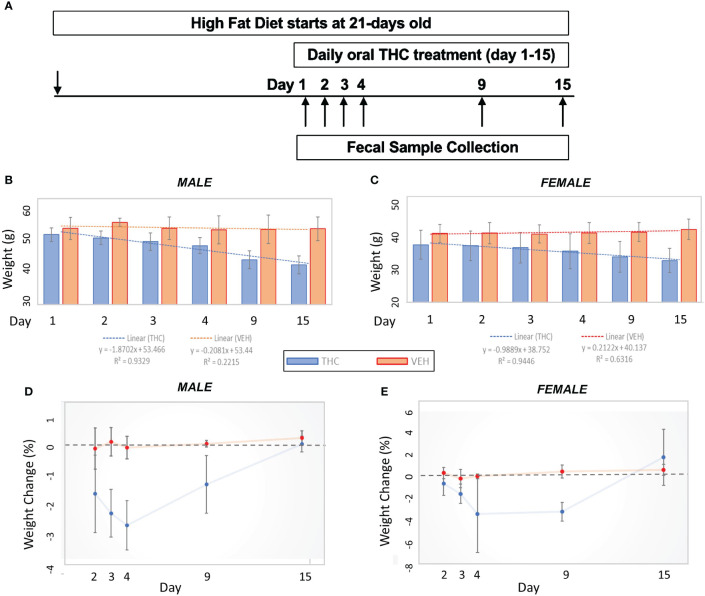
Weight change comparisons by treatment. **(A)** Experimental design flow chart. **(B, C)** Bar graph comparison in average weight over time by treatment in male (n=12) **(A)** and female (n=12) **(B)** mice. 95% confidence interval error bars displayed are in grams. Linear trend lines illustrate weight change over time with line statistics provided in the legend. **(D, E)** Daily weight change over time by treatment in male **(C)** and female **(D)** mice. 95% confidence interval error bars displayed are in percent weight change.

Mice were weighed daily, prior to drug administration. THC was formulated in sweetened condensed milk (Wild Harvest^®^ organic sweetened condensed milk, Eden Prairie, MN) at a ratio of 1:19. DIO mice were fed orally (p.o; not gavage) with a dose of THC (10 mg/kg) daily. Controls were fed the only “vehicle” (VEH) for drug delivery; henceforth, controls may be referred to as VEH. In the first cohort, there were 6 mice in each experimental group for each sex. The experiment lasted for two weeks. Percent weight change from baseline over the experimental duration (day=1 to day=15) was compared between THC and VEH using a two-tailed student’s t-test. The experimental design flow is outlined in **Figure 1A**.

### 2.3 Genomic DNA isolation

Fecal samples were collected on day=1, 2, 3, 4, 9 and 15 from each mouse (n=24) for a total of 142 samples (two samples were unable to be collected). Two to three fecal pellets were aseptically collected per animal and placed in individually labeled sterile tubes and 200 microliters of DNAse-free water was added. Samples were then stored at −80°C until processing. Fecal genomic DNA was extracted using the DNeasy PowerSoil (Qiagen) isolation kit. The protocol for DNA isolation provided by the manufacture was followed, with the exception that the initial vortex step was extended to 20 minutes to thoroughly homogenize the samples. The purified gDNA was quantified, as described above, and stored at −20°C in the supplied 10 mM Tris Buffer.

### 2.4 16S rRNA gene amplicon sequencing

The gDNA isolated from feces was used as the template for PCR amplification of the V4 variable region of the 16S rRNA gene sequence with region-specific primers (515F-816R). Amplicon sequencing was performed by the Institute for Genomics and Systems Biology at the Argonne National Laboratory (Argonne, IL) on the Illumina MiSeq Platform.

### 2.5 QIIME2 gut microbiome identification and composition analysis

Sequences were evaluated using the QIIME2 microbiome analysis platform version 2023.2 (Bolyen et al., 2019). The sequence data was demultiplexed and denoised utilizing DADA2 with the truncation parameters of f=250 and r=230 (Callahan et al., 2016). An example denoised output table from QIIME2 is provided in **Supplementary Figure 1**. Minimum feature frequency was set to 3. Sampling depth was set to 21,790 features for rarefaction, thereby removing 7/142 (4.9%) of fecal samples from the analysis.

Remaining sequences were aligned using the FastTree2 program to produce a rooted phylogenetic tree (Price et al., 2010).

Alpha diversity was analyzed with Shannon and Chao1 indices while beta diversity was investigated by applying weighted UniFrac distance (Lozupone et al., 2007). Difference in diversity between treatment groups were analyzed by pairwise ANOVA in QIIME2.

For the taxonomic analysis, feature sequences were assigned with q2-feature-classifier utilizing a classifier trained on the Greengenes 13_8 99% – which identifies bacterial taxonomic identity using 250 bases from the 16S gene region – to generate amplicon sequence variants (ASVs) using DADA2 (McDonald et al., 2012). Taxonomy bar-plots were produced at the phylum (2), class (3), order (4), family (5), genus (6) and species (7) taxonomic levels. Features are listed as p_phylum_c_class_o_order_f_family_g_genus_s_species. An example bar plot output from QIIME2 is provided in **Supplementary Figure 2**. Absolute abundance values were converted to relative abundance, thus normalizing data for subsequent analysis. We subsequently eliminated unnamed taxonomic features and features in less than 50% of samples.

Two additional QIIME2 plugins were utilized in the examination: 1) q2-sample-classifier, which attempts to predict metadata based on microbiome composition (Bokulich et al., 2018a); 2) q2-longitudinal, which inspects changes in alpha/beta diversity or metadata over time (Bokulich et al., 2018b).

For transparency, recorded information on the mice including weight and key bacterial relative abundance is provided in **Supplementary Tables 1** and **3** respectively for males and **Supplementary Tables 2** and **4** respectively for females. Full relative abundance tables can be provided upon request.

### 2.6 Differential gut microbiome analysis and modeling

Differences in gut microbiome composition and weight change modeling were performed using R (R Core Team, 2018). To investigate correlations between relative abundance, weight change and the effect of THC supplementation, linear mixed effects (LME) modeling was performed utilizing the lme4 package (Bates et al., 2015). LME overcomes several limitations of linear regression and ANOVA by accounting for baseline variability amongst individuals (Brown, 2021). Fixed slope random effects modeling is also recommended for experiments with smaller sample sizes and when predictor variance is anticipated to have similar impact on outcome variance between individuals; these parameters match our requirements (Harrison et al., 2018). Specialized LME R^2^ values were obtained with the r.squaredGLMM plugin command which produces two outputs: 1) Marginal R^2^ (R2M) which represents the variance explained by the fixed effects alone of the model and is ideal for comparing predictors; 2) Conditional R^2^ (R2C) which accounts for both the fixed and random effects and can be interpreted as variance explained by the complete model (Nakagawa et al., 2017). Bacterial features correlated with weight change were identified as weight change LME modes with a significant interaction between the feature relative abundance and timepoint.

Based on bacterial taxonomic features that both have significantly different abundance change between THC and VEH and predict weight change, LME models were generated to predict weight change based on bacterial relative abundance. To limit bias, the “best” models were chosen based on the combination of taxonomic features that yielded the highest R2C value regardless of potential biological explanation for including the specific features. Significance of individual LME models was evaluated using a likelihood ratio test (LRT) of model’s fit against the fit of a “null” LME model that only accounts for random effects of individual mice [null_model = lmer(weight_change ~ 1 + (1 | ratid)]. This essentially tests whether the predictive variables improve predictive value of the model beyond accounting for natural variation between individuals.

The “best” fit model was used to predict weight change in a second mouse cohort of both male (THC n=10, VEH n=6) and female (THC n=8, VEH n=3) mice using linear regression of predicted against actual weight change. In the second cohort, fecal samples were collected on day=1, 4, 9 and 15.

Plots were produced using the ggplot2 package in R (Wickham, 2016) or in QIIME2 (Bolyen et al., 2019).

## 3 Results

### 3.1 THC induces weight loss in obese mice

Over the 15-day duration of the experiment, in female mice, the THC-supplemented group (n=6) lost an average of 13.8% (95% CI −16.7% – −10.9%) of their body weight compared with an average gain of 2.9% (95% CI 0.9% – 4.9%) of body weight in controls (n=6) (t-test p<0.001). In male mice, the THC-treated group (n=6) lost an average of 17.8% (95% CI −14.8% – −20.8%) of their body weight compared with an average loss of 0.23% (95% CI −1.8% – 1.4%) of body weight in controls (n=6) (t-test p<0.001) (**Figures 1B, C**). The greatest amount of weight loss in the THC group tended to occur between days 3 and 9, especially in male mice, with a stabilization of weight by day 15 (**Figures 1D, E**).

### 3.2 Male and female mice microbiome composition differs at baseline

**Figure 2:**
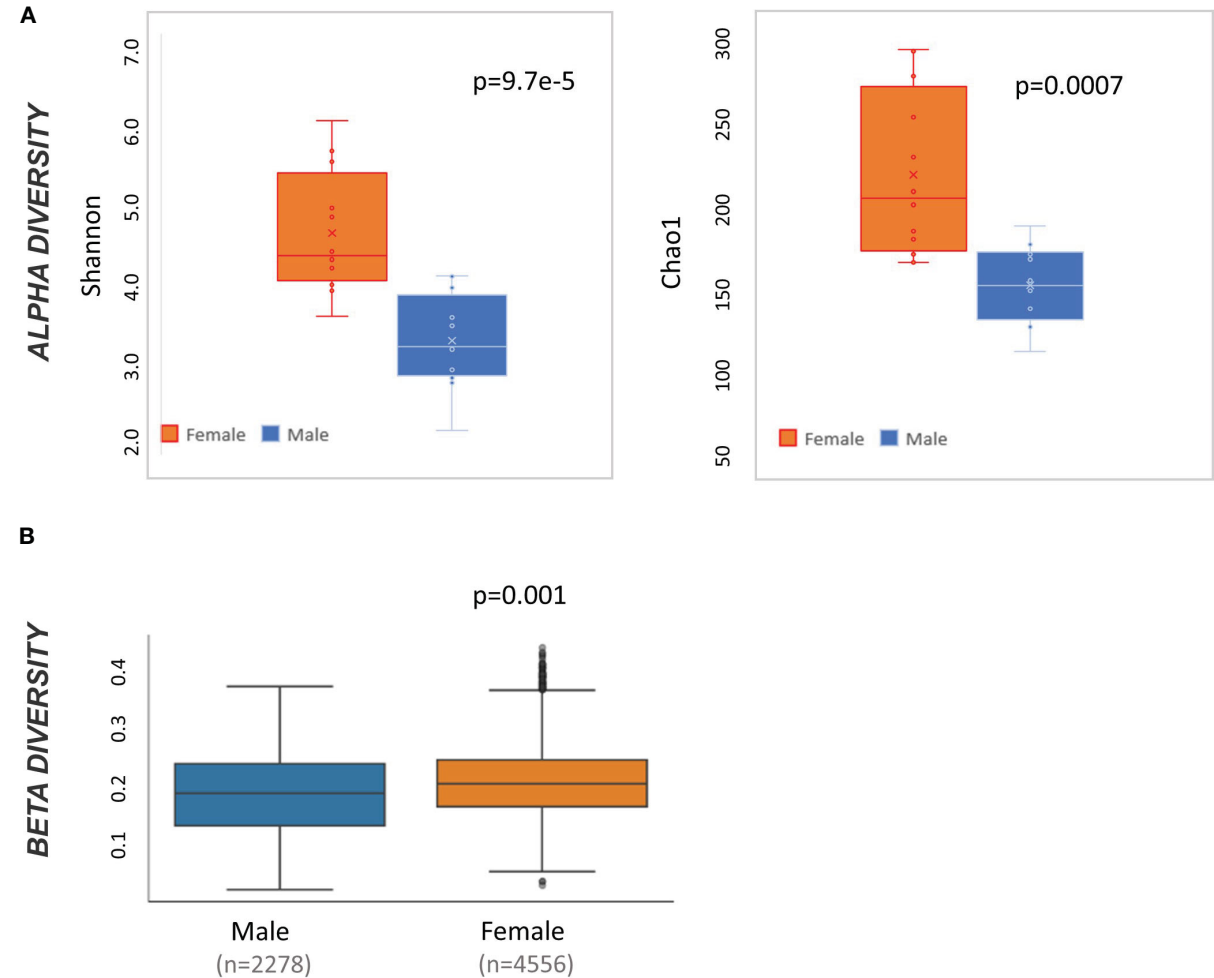
Microbiome diversity statistics at baseline by sex. **(A)** Box and whisker plot of alpha diversity values measured by Shannon and Chao1 and divided by sex. Result of t-test comparing male to female mice is displayed in the top right of each graph. **(B)** QIIME2-produced box and whisker plots of beta diversity values at baseline measured by weighted Unifrac and divided by sex. Result of pairwise ANOVA comparing meta to female mice is displayed in the bottom left of the plot.

At baseline, male and female mice have significantly different alpha diversity when measured by both Shannon (t-test p<0.001) and Chao 1 (t-test p<0.01) (**Figure 2A**). Likewise, weighted Unifrac beta diversity is also different at baseline by sex (pairwise ANOVA p=0.001) (**Figure 2B**). Accordingly, all subsequent taxonomic analysis were divided by sex.

### 3.3 Microbiome diversity analysis

**Figure 3:**
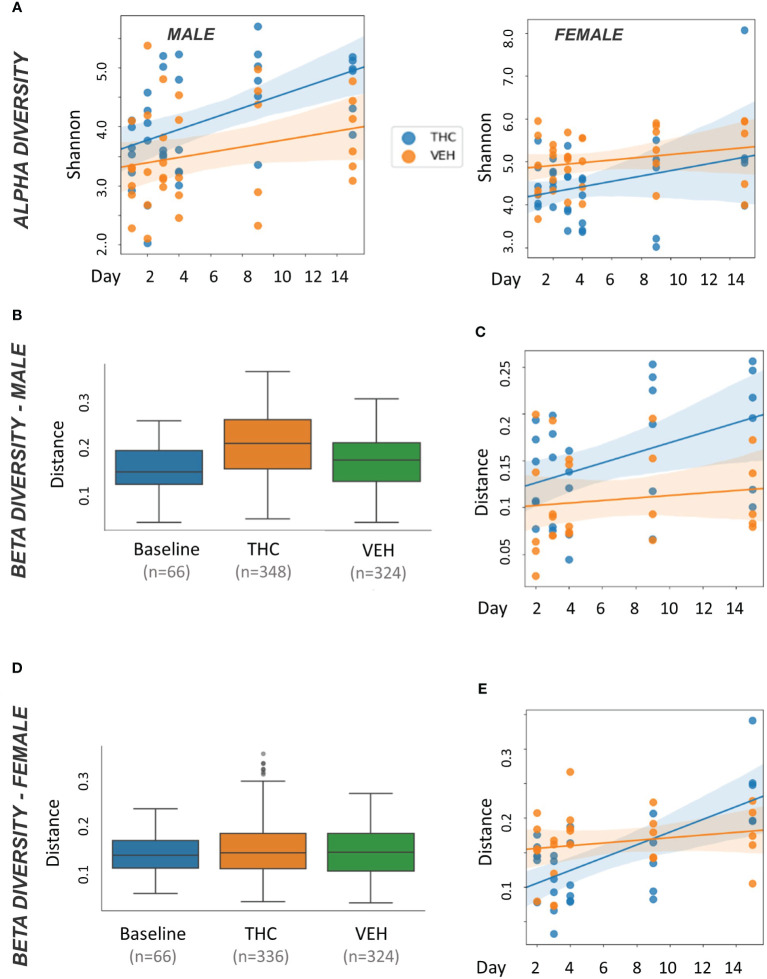
Microbiome diversity analysis. All plots in the figure were produced by QIIME2. **(A)** Scatter plot of alpha diversity values measured by Shannon divided by treatment for male (n=12) and female (n=12) mice. Trendlines are produced by LME model [Shannon ~ day × Q(treatment)] with 95% CI in shading. Pairwise ANOVA indicated a significant difference (p<0.05) in males on day 9 and in females (p<0.05) on days 2, 3 and 4. **(B, D)** Box and whisker plots of beta diversity measured by weighted Unifrac in male **(B)** and female **(D)** mice. THC and VEH groups are days 2–15 pooled. Significant beta diversity differences (p<0.05) by pairwise ANOVA were identified between baseline vs. THC and THC vs. VEH in males; females had a significant difference between THC vs. VEH. **(C, E)** Scatter plot of beta diversity values measured by weighted Unifrac and divided by treatment in male **(C)** and female **(E)** mice. Trendlines are produced by first distances LME models [Distance ~ day × Q(treatment)] with 95% CI in color shading. Males had a significant difference (p<0.05) on day 9.

In male mice, THC supplementation increased Shannon alpha diversity with a significant difference by day 9 (p<0.05) (**Figure 3A**). However, the trend was different in female mice with a convergence in alpha diversity between treatment groups (**Figure 3A**). For male mice, with all treatment days (days 2–15) pooled, there was a significant difference (p<0.05) in beta diversity between THC-treated and baseline in addition to THC-treated and VEH (**Figure 3B**). When separated by day, there was only a significant difference (p<0.05) in beta diversity between treatment groups on day 9 (**Figure 3C**). In female mice, with all treatment days pooled, there was a significant difference in beta diversity between THC and VEH mice (p<0.05) (**Figure 3D**). However, when separated by day, there was no individual timepoint with a significant difference (**Figure 3E**).

### 3.4 Taxonomic analysis in male mice

**Figure 4:**
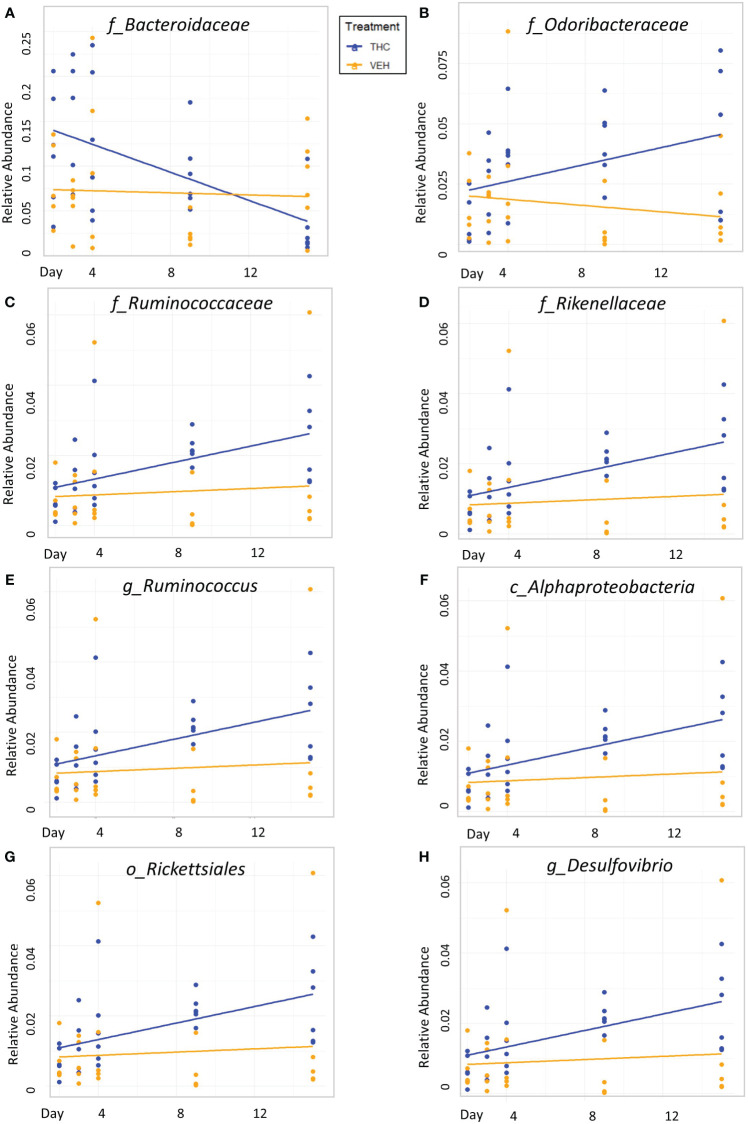
Bacterial relative abundance change over time comparison by treatment in male mice. Scatter plots displaying the relative abundance of 8 individual bacterial taxonomic features (identified at the top of each panel **A–H**) over time (Day) with treatment group identified by color. The trendlines are linear regressions of relative abundance over time separated by treatment. All 8 bacterial features have significantly different LME slopes between THC (n=6) and VEH (n=6).

**Figure 5:**
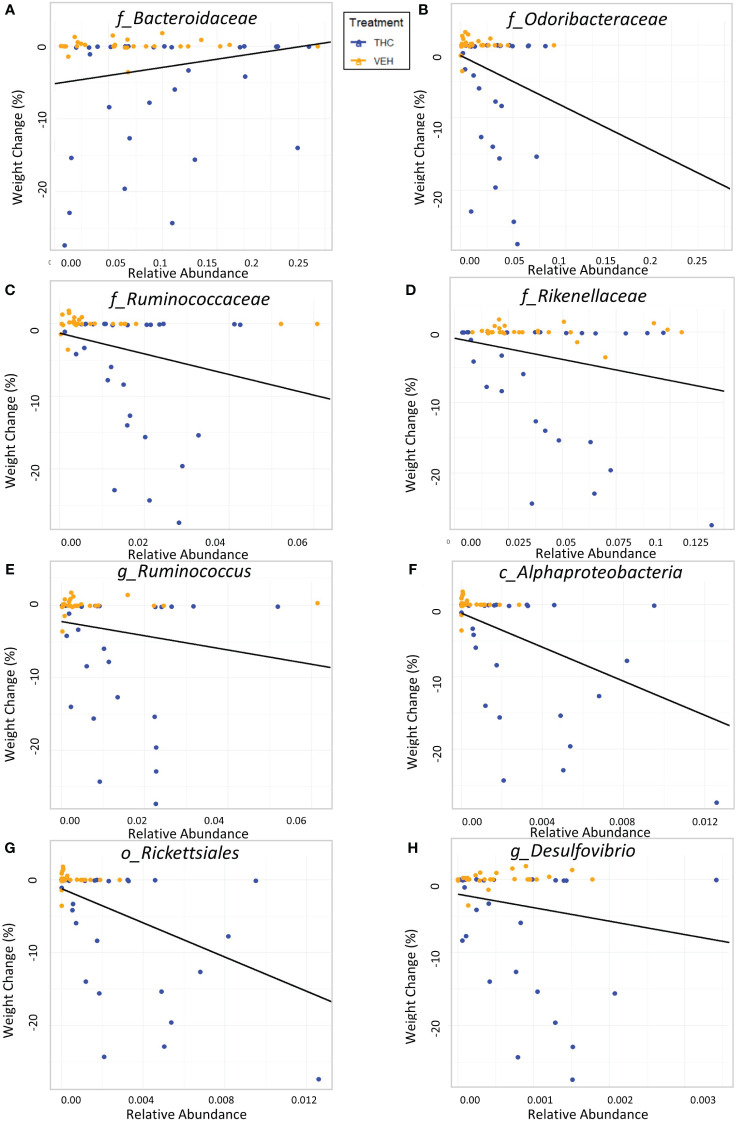
Relationship between percent weight change from baseline and bacterial relative abundance in male mice. Scatterplots displaying the percent weight change from baseline against relative abundance for 8 individual taxonomic features (identified at the top of each panel **A–H**). Plot points are separated by treatment and indicated by color. Trendline is the result of a significant LME modeling of percent weight change against relative abundance.

Due to baseline microbiome composition differences, taxonomic analysis was performed separately for male and female mice. In male mice, there were 24 bacterial taxonomic features with significant (p<0.05) differences in relative abundance change over the experimental duration between THC and VEH based on LME models (**Table 1**). There were also 24 bacterial taxonomic features with significant LME models predicting weight change over time (**Table 2**). 8 of the bacterial features, listed in **Table 3** for convenience, were both significantly impacted by THC supplementation (**Figure 4**) and correlated with weight change (**Figure 5**). 7 of the 8 bacterial features identified displayed an inverse relationship between relative abundance and percent weight change from baseline (**Table 4**; **Figure 5**). Combinations of the 8 bacterial taxonomic features were subsequently utilized to produce a combined LME model with the strongest predictive value of weight change independent of timepoint and final treatment.

**Table 1:** Bacterial taxonomic features with significantly different change in relative abundance between THC and VEH in male mice.

Taxon	p-value	THC change (%)	VEH change (%)
*Bacteroidetes; Bacteroidia; Bacteroidales; Bacteroidaceae*	*	−7.785%	−0.283%
*Bacteroidetes; Bacteroidia; Bacteroidales; Odoribacteraceae*	**	3.281%	0.268%
*Bacteroidetes; Bacteroidia; Bacteroidales; Odoribacteraceae; Odoribacter*	*	2.758%	0.233%
*Bacteroidetes; Bacteroidia; Bacteroidales; Prevotellaceae; Prevotella*	*	0.013%	−0.012%
*Bacteroidetes; Bacteroidia; Bacteroidales; Rikenellaceae*	*	1.578%	0.540%
*Bacillota; Bacilli; Lactobacillales; Lactobacillaceae; Lactobacillus; salivarius*	*	0.429%	3.080%
*Bacillota; Clostridia; Clostridiales; Peptococcaceae*	*	0.152%	−0.004%
*Bacillota; Clostridia; Clostridiales; Ruminococcaceae*	**	5.585%	2.225%
*Bacillota; Clostridia; Clostridiales; Ruminococcaceae; Ruminococcus*	**	1.678%	0.314%
*Proteobacteria; Alphaproteobacteria*	***	0.464%	0.019%
*Proteobacteria; Alphaproteobacteria; Rickettsiales*	***	0.465%	0.021%
*Proteobacteria; Betaproteobacteria*	*	−0.804%	0.070%
*Proteobacteria; Betaproteobacteria; Burkholderiales*	*	−0.804%	0.074%
*Proteobacteria; Betaproteobacteria; Burkholderiales; Alcaligenaceae*	*	−0.810%	0.071%
*Proteobacteria; Betaproteobacteria; Burkholderiales; Alcaligenaceae; Sutterella*	**	−1.080%	0.035%
*Proteobacteria; Deltaproteobacteria; Desulfovibrionales; Desulfovibrionaceae; Bilophila*	*	0.038%	0.006%
*Proteobacteria; Deltaproteobacteria; Desulfovibrionales; Desulfovibrionaceae; Desulfovibrio*	*	0.095%	0.015%
*Proteobacteria; Epsilonproteobacteria*	*	0.048%	1.974%
*Proteobacteria; Epsilonproteobacteria; Campylobacterales*	*	0.048%	1.974%
*Proteobacteria; Epsilonproteobacteria; Campylobacterales; Helicobacteraceae*	*	0.048%	1.974%
*Tenericutes*	***	1.543%	−0.439%
*Tenericutes; Mollicutes*	***	1.543%	−0.439%
*Tenericutes; Mollicutes; Mycoplasmatales*	***	1.511%	−0.419%
*Tenericutes; Mollicutes; Mycoplasmatales; Mycoplasmataceae*	***	1.511%	−0.419%

Individual bacterial taxonomic features were modeled using the LME: lmer([taxon] ~ day × treatment + (1 | ratid)). Features listed on the table had a significantly different slope of the LME model (indicating difference in relative abundance change) between THC (n=6) and VEH (n=6). The model p-value refers to the interaction treatment × day. THC and VEH percent relative abundance change over the 15-day experiment are also listed. [* = *p*<0.05, ** = *p* <0.01, *** = *p*<0.001].

**Table 2:** Bacterial taxonomic features with significant models predicting weight change over time in male mice.

Taxon	p-value	Coefficient
*Bacteroidetes; Bacteroidia; Bacteroidales; Bacteroidaceae*	*	22.50616
*Bacteroidetes; Bacteroidia; Bacteroidales; Bacteroidaceae; Bacteroides*	*	18.77231
*Bacteroidetes; Bacteroidia; Bacteroidales; Odoribacteraceae*	*	−71.84106
*Bacteroidetes; Bacteroidia; Bacteroidales; Rikenellaceae*	*	−142.04226
*Bacteroidetes; Bacteroidia; Bacteroidales; Rikenellacea; Alistipes; indistinctus*	*	9185.39929
*Deferribacteres*	**	−128.88402
*Deferribacteres; Deferribacteres*	**	−128.88402
*Deferribacteres; Deferribacteres; Deferribacterales*	**	−128.88402
*Deferribacteres; Deferribacteres; Deferribacterales; Deferribacteraceae*	**	−128.88402
*Deferribacteres; Deferribacteres; Deferribacterales; Deferribacteraceae; Mucispirillum*	*	−80.74148
*Bacillota; Bacilli; Lactobacillales; Enterococcaceae*	***	729.02815
*Bacillota; Bacilli; Lactobacillales; Enterococcaceae; Enterococcus*	**	630.35377
*Bacillota; Bacilli; Lactobacillales; Lactobacillaceae*	*	32.5253
*Bacillota; Bacilli; Lactobacillales; Lactobacillaceae; Lactobacillus*	*	32.24164
*Bacillota; Clostridia; Clostridiales; Mogibacteriaceae*	*	1269.81785
*Bacillota; Clostridia; Clostridiales; Ruminococcaceae*	*	−60.01989
*Bacillota; Clostridia; Clostridiales; Ruminococcaceae; Ruminococcus*	**	−98.32073
*Proteobacteria*	**	−38.84509
*Proteobacteria; Alphaproteobacteria*	***	−1176.60235
*Proteobacteria; Alphaproteobacteria; Rickettsiales*	***	−1174.71325
*Proteobacteria; Deltaproteobacteria*	**	−45.59683
*Proteobacteria; Deltaproteobacteria; Desulfovibrionales*	**	−45.61599
*Proteobacteria; Deltaproteobacteria; Desulfovibrionales; Desulfovibrionaceae*	**	−45.61599
*Proteobacteria; Deltaproteobacteria; Desulfovibrionales; Desulfovibrionaceae; Desulfovibrio*	*	−1840.782

Individual bacterial taxonomic features were used to predict weight change using the LME: lmer(weight_change ~ Q[[taxon]] + (1 | ratid)). Features with a statistically significant correlation between relative abundance and weight change are listed along with the p-value for the correlation and the model coefficient (Q). [* = *p*<0.05, ** = *p*<0.01, *** = *p*<0.001].

**Table 3:** List of bacterial taxonomic features that both predict weight change and had significant difference in relative abundance change between THC and VEH in male mice.

Key bacterial taxonomic features in male mice
•*Bacteroidetes; Bacteroidia; Bacteroidales; Bacteroidaceae (Bacteroidaceae)*
•*Bacteroidetes; Bacteroidia; Bacteroidales; Odoribacteraceae (Odoribacteraceae)*
•*Bacteroidetes; Bacteroidia; Bacteroidales; Rikenellaceae (Rikenellaceae)*
•*Bacillota; Clostridia; Clostridiales; Ruminococcaceae (Ruminococcaceae)*
•*Bacillota; Clostridia; Clostridiales; Ruminococcaceae; Ruminococcus (Ruminococcus)*
•*Proteobacteria; Alphaproteobacteria (Alphaproteobacteria)*
•*Proteobacteria; Alphaproteobacteria; Rickettsiales (Rickettsiales)*
•*Proteobacteria; Deltaproteobacteria; Desulfovibrionales; Desulfovibrionaceae; Desulfovibrio (Desulfovibrio)*

**Table 4:** LME models of percent weight change from baseline against bacterial relative abundance for eight individual taxonomic features in male mice.

Bacterial Taxonomic Feature	p-value	Coefficient	R2M | R2C
*Bacteroidetes; Bacteroidia; Bacteroidales; Bacteroidaceae*	0.0322	22.506	0.0402 | 0.7069
*Bacteroidetes; Bacteroidia; Bacteroidales; Odoribacteraceae*	0.0255	−71.841	0.0491 | 0.6821
*Bacteroidetes; Bacteroidia; Bacteroidales; Rikenellaceae*	0.012	−142.042	0.0674 | 0.7009
*Bacillota; Clostridia; Clostridiales; Ruminococcaceae*	0.00291	−60.0199	0.0603 | 0.7068
*Bacillota; Clostridia; Clostridiales; Ruminococcaceae; Ruminococcus*	0.0426	−98.321	0.0301 | 0.6853
*Proteobacteria; Alphaproteobacteria*	2.14e-6	−1176.602	0.2171 | 0.7330
*Proteobacteria; Alphaproteobacteria; Rickettsiales*	2.11e-6	−1174.713	0.2182 | 0.7326
*Proteobacteria; Deltaproteobacteria; Desulfovibrionales; Desulfovibrionaceae; Desulfovibrio*	0.0491	−1840.702	0.0300 | 0.6671

Model statistics for the LME of individual taxonomic features [lmer(weight_change ~ Q[[taxon]] + (1 | ratid))]. Includes p-value for the model, coefficient (Q), marginal R^2^ (R2M) and conditional R^2^ (R2C).

### 3.5 Building a 3-feature LME model to predict weight change using specific bacterial taxonomic feature relative abundance

The next goal was to investigate the utility of modeling weight change only utilizing bacterial features significantly altered by THC supplementation and compare to an ideal weight change model which accounts for treatment (THC or VEH) and timepoint (**Figure 1C**). Out of all combinations of bacterial taxonomic features in male mice, the model with the highest predictive value for weight change of the fixed effects (i.e., R2M), henceforth called the “3-feature model,” contained three: *p_Bacteroidetes_c_Bacteroidia_o_Bacteroidales_f_Rikenellaceae* (*Rikenellaceae*), *p_Bacillota_c_Clostridia_o_Clostridiales_f_Ruminococcaceae* (*Ruminococcaceae*) and *p_Proteobacteria_c_Alphaproteobacteria_o_Rickettsiales* (*Rickettsiales*).


Male-Produced 3-Feature Model:Weight_Change(%)∼−77.2±95.6×[Rikenellaceae]+−1007.6±501.8×[Rickettsiales]+−9.6±39×[Ruminococcaceae]−0.1±3.3+(1|ratid)

The fixed effects of the bacterial features in the 3-feature model explained 23.7% of the variation in weight change alone while the final model, which includes random effects of individual mice, yielded an R2C of 0.75 (**Supplementary Figure 3A**). Furthermore, the model’s fit was significantly greater than the null model (chi-squared p<0.001), confirming that the addition of bacterial features improved prediction of weight change beyond accounting for random effect of individual mice (**Supplementary Figure 3A**). The 3-feature model explained more variation in weight change than one only accounting for final treatment, THC or VEH (20.5%) (**Supplementary Figure 3B**). Adding final treatment to the 3-feature model fixed effects increased the percent variation explained to 36.3% and yielded a final R2M of 0.79 (**Supplementary Figure 3C**). When investigating the effect of timepoint (day) on weight change, the addition to fixed effects of the original 3-feature model only marginally increased the R2M (0.25) and R2C (0.77) (**Supplementary Figure 3D**). The fixed effect of timepoint alone was very poor at explaining variation in weight change with an R2M of 0.06 (**Supplementary Figure 3F**). The ideal LME model explained 31.2% of the variation in weight change with the fixed effects and yielded an R2M of 0.80 (**Supplementary Figure 3E**). Therefore, the fixed effects of the male-produced 3-feature model reached 76% of the predictive value of the optimal model by simply utilizing the relative abundance of three bacterial taxonomic features.

### 3.6 Testing the 3-feature model on a second experimental cohort of male mice

**Figure 6:**
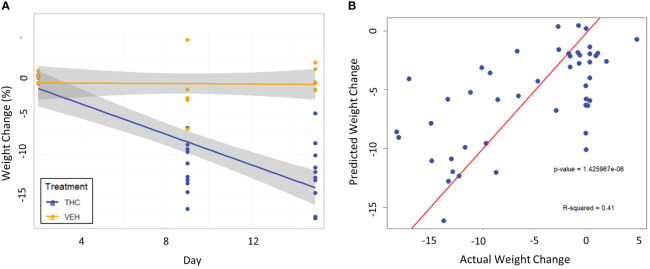
Testing the 3-feature LME model on a second cohort of male mice. **(A)** Scatter plot and linear regression lines of percent weight change from baseline over time divided by treatment – VEH (n=10) and VEH (n=6). 95% confidence intervals in gray shading. **(B)** Scatterplot of predicted weight change percentage based on the 3-feature model against actual weight change in the second mouse cohort. Linear regression line is superimposed on the plot with the associated p-value and R^2^ for the regression.

To test the validity of the 3-feature model, the exact same experimental protocol was repeated on a second cohort of male mice. A different group of mice will naturally be exposed slightly different environmental conditions and differ genetically; therefore, their gut microbiome composition will be different than the original cohort. Again, the THC cohort (n=10) had significant weight loss compared to controls (n=6) with the mice losing 12.5% (95% CI −10.1% – −14.9%) and 0.41% (95% CI −1.47% – 0.61%) respectively (t-test p<0.001) (**Figure 6A**). A large 71.7% of variation in weight change was explained by treatment and temporal effects in LME modeling (**Supplementary Figure 4A**). When the 3-feature model was applied to the second cohort, the fixed effects from relative abundance of the bacterial features explained 39.0% of the variation in weight change and was a significantly greater fit than the null model (chi-squared p<0.001) (**Figure 6B**). There was also a significant correlation between predicted weight change from the male 3-feature model against actual weight change in the second mouse cohort (Pearson correlation R=0.64, R^2^=0.41, p<0.001) (**Supplementary Figure 4B**).

### 3.7 Taxonomic analysis and weight change modeling in female mice

**Figure 7:**
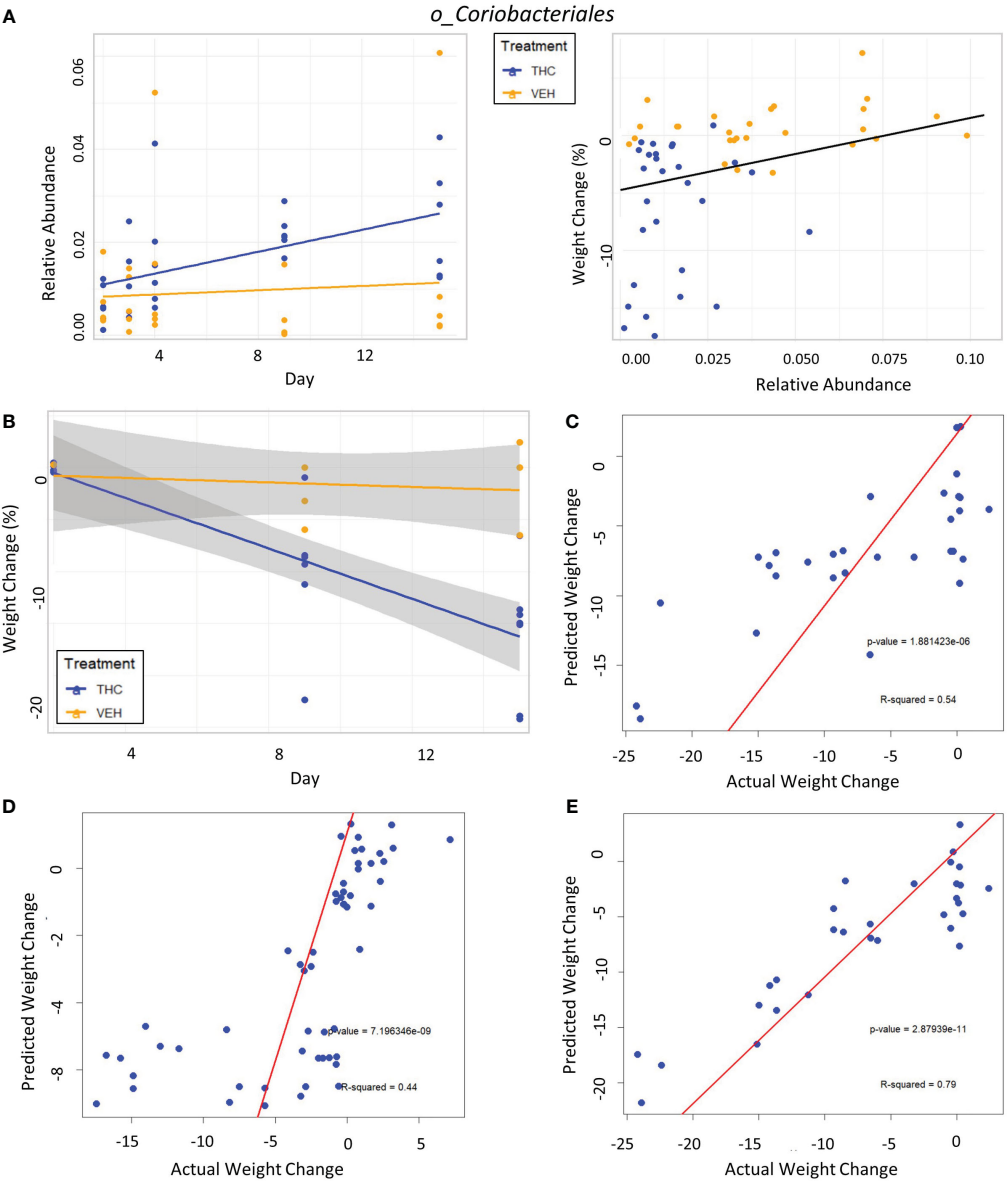
Taxonomic analysis and testing bacterial feature-based LME weight change models in female mice. **(A)** Left scatter plot displays the relative abundance of a single bacterial taxonomic feature over time with treatment group identified by color. The trendline is a linear regression of relative abundance over time separated by treatment with the corresponding LME equation slope, p-value and R^2^ matched by color. Right scatterplot displays the percent weight change from baseline against relative abundance for an individual taxonomic feature. Plot points are separated by treatment and indicated by color. Trendline is the result of an LME modeling percent weight change with change in relative abundance. The number on the bottom right is the p-value for the LME model. **(B)** Scatterplot of percent weight change over time (day) with linear trendlines for THC (n=6) and VEH (n=6) groups indicated by color. Grey shading is 95% confidence interval with value of percent weight change. **(C–E)** Each panel is a scatterplot of predicted weight change percentage based on the respective 3-feature LME model against actual weight change in female mice. Linear regression line is superimposed on the plot with the associated p-value and R^2^ for the regression. **(C)** Testing the female-specific 3-feature LME model in the second cohort of female mice (THC n=8, VEH n=3). **(D)** Testing the male-produced 3-feature LME model in the original female mice cohort. **(E)** Testing the male-produced 3-feature LME model in the second female mice cohort.


In female mice, there were 14 bacterial taxonomic features with significant differences in relative abundance change over the experimental duration between THC (n=6) and VEH (n=6) based on LME models (**Table 5**). There were also 12 bacterial taxonomic features with significant LME models predicting weight change over time (**Table 6**). Six of the bacterial features were both significantly impacted by THC supplementation and correlated with weight change (**Table 7**) and none of which matched a key feature identified in the male mice. Upon visual analysis of the scatterplots relating bacterial relative abundance to weight change, only one significantly impacted by THC, *p_Actinobacteria_c_Coriobacteriia_o_Coriobacteriales* (*Coriobacteriales*), was not drastically impacted by outliers (**Figure 7A**). Producing an effective LME weight change model that explained a significant portion of the variance of timepoint and treatment (**Supplementary Figure 5C**) was more difficult in the female mice than in male mice. The best model in females also contained three bacterial features: *p_Actinobacteria_c_Actinobacteria_o_Actinomycetales* (*Actinomycetales*), *p_Actinobacteria_c_Actinobacteria_o_Coriobacteriales* (*Coriobacteriales*) and *p_Bacillota_c_Bacilli_o_Lactobacillales_f_Lactobacilliaceae_g_Lactobacillus_s_Salivarius* (L. *salivarius*), but only explained 14.6% of the variance in weight change, yielded an R2C of 0.357, and was only marginally more significant of a fit than the null model (p=0.02) (**Supplementary Figure 5A**).

**Table 5:** Bacterial taxonomic features with significantly different change in relative abundance between THC and VEH in female mice.

Taxon	p-value	THC change (%)	VEH change (%)
*Actinobacteria; Actinobacteria*	*	2.2471%	0.0002%
*Actinobacteria; Actinobacteria; Actinomycetales*	*	2.2489%	0.0002%
*Actinobacteria; Coriobacteriia*	*	−1.3855%	2.4996%
*Actinobacteria; Coriobacteriia; Coriobacteriales*	*	−1.3855%	2.4996%
*Actinobacteria; Coriobacteriia; Coriobacteriales; Coriobacteriaceae*	*	−1.3855%	2.4996%
*Bacillota; Bacilli; Lactobacillales; Lactobacillaceae*	*	0.4593%	2.8856%
*Bacillota; Bacilli; Lactobacillales; Lactobacillaceae; Lactobacillus*	*	0.4585%	2.8845%
*Bacillota; Bacilli; Lactobacillales; Lactobacillaceae; Lactobacillu; salivarius*	*	−0.2430%	0.8041%
*Bacillota; Clostridia; Clostridiales; Lachnospiraceae; Coprococcus*	*	1.0932%	−0.1847%
*Tenericutes; Mollicutes; RF39*	*	0.1270%	−0.0250%
*TM7*	**	0.1066%	−0.0162%
*TM7; TM7*	**	0.0186%	−0.0162%
*TM7; TM7_3; CW040*	*	0.0120%	−0.0162%
*TM7; TM7_3; CW040_f:F16*	*	0.0120%	−0.0162%

Individual bacterial taxonomic features were modeled using the LME: lmer([taxon] ~ day × treatment + (1 | ratid)). Features listed on the table had a significantly different slope of the LME model (indicating difference in relative abundance change) between THC (n=6) and VEH (n=6). The model p-value refers to the interaction treatment × day. THC and VEH percent relative abundance change over the 15-day experiment are also listed. [* = *p*<0.05, ** = *p*<0.01, *** = *p*<0.001].

**Table 6:** Bacterial taxonomic features with significant models predicting weight change over time in female mice.

Taxon	p-value	Coefficient
*Actinobacteria; Actinobacteria*	*	−96.1873
*Actinobacteria; Actinobacteria; Actinomycetales*	*	−96.2001
*Actinobacteria; Coriobacteriia*	*	62.75089
*Actinobacteria; Coriobacteriia; Coriobacteriales*	*	62.75089
*Actinobacteria; Coriobacteriia; Coriobacteriales; Coriobacteriaceae*	*	62.75089
*Bacteroidetes; Bacteroidia; Bacteroidales; Porphyromonadaceae*	*	94.89083
*Bacteroidetes; Bacteroidia; Bacteroidales; Porphyromonadaceae; Parabacteroides*	*	94.90096
*Bacillota; Bacilli; Lactobacillales; Lactobacillaceae; Lactobacillus; salivarius*	*	173.8052
*Bacillota; Clostridia; Clostridiales; Mogibacteriaceae*	**	3341.389
*Tenericutes*	*	−464.394
*Tenericutes; Mollicutes*	*	−464.394
*TM7*	*	−2349.97

Individual bacterial taxonomic features were used to predict weight change using the LME: lmer(weight_change ~ Q[[taxon]] + (1 | ratid)). Features with a statistically significant correlation between relative abundance and weight change are listed along with the p-value for the correlation and the model coefficient (Q). [* = *p*<0.05, ** = *p*<0.01, *** = *p*<0.001].

**Table 7:** LME models of percent weight change from baseline against bacterial relative abundance for six individual taxonomic features in female mice.

Bacterial Taxonomic Feature	p-value	Coefficient	R2M | R2C
*Actinobacteria; Actinobacteria*	0.026	−96.187	0.0657 | 0.4173
*Actinobacteria; Actinobacteria; Actinomycetales*	0.0267	−96.20	0.0655 | 0.4172
*Actinobacteria; Coriobacteriia*	0.0421	62.751	0.0765 | 0.2686
*Actinobacteria; Coriobacteriia; Coriobacteriales*	0.0421	62.751	0.0765 | 0.2686
*Actinobacteria; Coriobacteriia; Coriobacteriales; Coriobacteriaceae*	0.0421	62.751	0.0765 | 0.2686
*Firmicutes; Bacilli; Lactobacillales; Lactobacillaceae; Lactobacillus; salivarius*	0.0491	172.805	0.0614 | 0.3696

Model statistics for the LME of individual taxonomic features [lmer(weight_change ~ Q[[taxon]] + (1 | ratid))]. Includes p-value for the model, coefficient (Q), marginal R^2^ (R2M) and conditional R^2^ (R2C).

Female-Produced 3-Feature Model:Weight_Change(%)=−87±83.7×[Actinomycetales]+38.8±62.3×[Coriobacteriales]+114.7±182.1[L. Salivarius]−5.1±2.7

The addition of timepoint substantially improved the model with fixed effects explaining 33.1% of variation in weight, thus indicating that other changes occurring during the experimental duration outside those microbiome features better account for body mass changes (**Supplementary Figure 5B**).

In the second mouse cohort females, as observed in male mice and in the first female cohort, THC (n=8) induced significant weight loss in 2 weeks compared with VEH (n=3) (**Figure 7B**), generating an average weight loss of 15.8% (95% CI −12% – −19.4%) and 1.3% (95% CI −5.6% – 3%) body weight respectively (t-test p<0.001). The female-generated 3-feature model better explained variation in the second female cohort with an R2M of 0.319, was a significantly greater fit than the null model (p<0.001) (**Supplementary Figure 6B**) and accurately predicted weight change (Pearson correlation R=0.73, R^2^=0.54, p<0.001) (**Figure 7C**). The 3-feature model produced from the male mice was very poor at explaining variation in weight change in the original female mice cohort (**Supplementary Figure 6C**). But interestingly, when comparing predicted weight change from the male-produced 3-feature model against actual weight change in the female mice using linear regression, the model’s predictions still significantly correlated with the real-world results (Pearson correlation R=0.66, R^2^=0.44, p<0.001) (**Figure 7D**). Surprisingly, the male-generated 3-feature model described a large variation in weight change for the second female cohort (**Supplementary Figure 6D**), had the most accurate weight change prediction (Pearson correlation R=0.89, R^2^=0.79, p<0.001) and profoundly greater fit than the null model (p<0.001) (**Figure 7E**).

## 4 Discussion

The goal of our present investigation was to determine the efficacy of THC supplementation in reducing weight in obese mice and to reveal potential contributions of gut microbiome changes to the THC-induced body mass decrease.

### 4.1 THC supplementation induces weight loss in obese mice regardless of sex

While previous research demonstrated that low-dose THC administration can prevent weight gain in DIO mice (Cluny et al., 2015), our current experimental data strongly support the hypothesis that adding THC to the diet will initiate weight loss in the obese animal model. The effects held for both males and females with 17.8% and 13.8% decreases in body weight respectively in two weeks compared with no change in weight for controls. The greatest degree of weight loss tended to occur between days 4 and 9 from intervention onset with a stabilization by day 15. The same trend occurred in a second cohort of male and female mice. It has been proposed that exogenous low efficacy eCB1 agonists like THC can reduce the binding of lower affinity, high efficacy CB1 agonists like 2-AG in high eCB tone conditions like obesity (Le Foll et al., 2013). Returning ECS activity towards equilibrium will thus theoretically reduce inflammation, improve gut barrier function and reduce adipogenesis (Cuddihey et al., 2022). There is also increasing evidence of a microbiota-to-adipose tissue regulatory loop with reciprocal effects on ECS tone (Muccioli et al., 2010). Therefore, our research focus turned towards answering whether THC administration altered gut microbiome composition and if those changes potentially contribute to weight change.

### 4.2 Approach to taxonomic analysis and determining THC-induced changes

We implemented a step-wise approach to select bacterial features of interest. Upon taxonomic analysis, we identified sex-specific gut microbiome changes of distinct bacterial taxonomic features that are both significantly impacted by THC administration and correlated with weight change. Initially, we observed that male and female mice had different gut microbiome composition at baseline post-DIO protocol, paralleling past research into sex-based differences in gut microbiota in response to high-fat diet (Bridgewater et al., 2017; Martin et al., 2017). Furthermore, there is evidence of brain region variances in eCB levels between males and females (Rubino and Parolaro, 2011), sex-dependent effects of THC use on brain function (Cooper and Craft, 2018), and sex differences in interactions between hormones, the immune system and gut microbiota (Vemuri et al., 2019). It is therefore not surprising that THC differentially affected microbiota in males and females. Our data further stress the importance of considering sex when investigating gut microbiome composition and obesity.

We subsequently utilized bacterial taxonomic features whose abundance significantly changes between THC and VEH and correlates with weight change to generate models to predict percent weight change based solely on bacterial relative abundance. We decided to utilize LME modeling as opposed to linear regression or two-way ANOVA due to its ability to account for natural baseline inter-individual variation and correlation between observations by distinguishing random from fixed effects (Murphy et al., 2022). To limit bias, the final models were chosen based on the combination of bacterial taxonomic feature predictors that explain the largest degree of variation in weight change (i.e., highest R2M value) without considering biological explanation for their inclusion.

### 4.3 Microbiome-based weight change modeling in male mice

The LME “3-feature” weight change model produced from male mice included *Ruminococcaceae*, *Rikenellaceae* and *Rickettsiales* – all of which inversely correlated with weight – and alone explained 23.7% of variation in weight change. Furthermore, the model was profoundly more accurate than a “null” model that only accounts for random effects of individual mice (p<0.001). For comparison, an idealized model utilizing final treatment and timepoint explained 31.2% of the variation, meaning that our 3-feature model reached 76% the predictive value of that idealized model.

To validate our model, we proceeded to test it on a second cohort of mice that followed the same experimental protocol. This analysis assessed external validity since the baseline microbiome of a different group of mice will have some dissimilarities simply due to environmental differences. The 3-feature model successfully predicted weight change in the second cohort (R=0.64, p<0.001) and explained an even larger fraction (39.1%) of the variation in weight change.

Despite avoiding artificial selection of predictors based on biological reasoning, most of the bacterial features identified as significant contributors in the 3-feature model have previously known relationships with metabolism and weight regulation. Regarding *Ruminococcaceae*, lower abundances have been correlated with diabetes mellitus (Bajaj et al., 2015), obesity (Feng et al., 2022), and high-fat diets (Daniel et al., 2014) with increased abundance in lean mice (Feng et al., 2022). *Ruminococcaceae* have known involvement in butyrate production (Daniel et al., 2014) and 7α de-hydroxylation – the conversion of primary to secondary bile acids (Kakiyama et al., 2013). Interestingly, a human study found lower abundance of *Ruminococcaceae* in patients with cirrhosis and a negative correlation with *Bacteroidaceae* which paralleled an observation in our study (Kakiyama et al., 2013). The relationship we detected between increased *Rikenellaceae* and greater weight loss also has sound biological reasoning. Reduction in *Rikenellaceae* is associated with increased adiposity (Vallianou et al., 2021) and reduced brown adipose tissue (Guzzardi et al., 2022). Conversely, increased abundance corresponds to reduced visceral adipose tissue (Tavella et al., 2021) and greater lipid metabolism along with *Ruminococcaceae* (Conte et al., 2022). It is less clear why the order *Rickettsiales* was consistently detected as a significant predictor for weight loss. *Rickettsiales* are obligate intracellular parasites with limited oxidative metabolism capabilities; accordingly, their transport systems likely impact host metabolite availability (Driscoll et al., 2017). While most known genera in *Rickettsiales* are associated with pathological conditions, there is some evidence of specific species participating in positive endosymbiosis with hosts through amino acid anabolism (Carrier et al., 2021), decreased inflammation in IBD models (Liu et al., 2020), and fatty acid or glycerophospholipid utilization (Driscoll et al., 2017). While we cannot rule out chance in our consistent *Rickettsiales* detection, it is certainly possible there are potentially metabolically beneficial species within the order. The fact that our agnostic approach to feature selection mostly detected bacteria associated with metabolism strengthens the validity of the modeling method.

It is worthy of note that the second mouse cohort had a strong correlation between g_Akkermansia _s_muciniphilia (*A. muciniphilia*) and weight loss, a species with known roles in glucose and lipid metabolism (Zhang et al., 2019; Xu et al., 2020) and an inverse relationship with obesity and type 2 diabetes (Everard et al., 2013; Remley et al., 2016). However, there was no *A. muciniphilia* detected at all in the first cohort, so it was not included in the 3-feature model. Our observation accentuates the marked influence environmental exposures have on gut microbiome (Ahn and Hayes, 2021). Therefore, baseline variability must be carefully considered in any interpretation of microbiome research on disease models (He et al., 2018).

### 4.4 Microbiome-based weight change modeling in female mice

Weight change modeling proved to be much more difficult in female mice. It is established that sex hormones have differential effects on gut microbiome composition (Org et al., 2016; McGee and Huttenhower, 2021) including a bi-directional estrogen-microbiome axis (Baker et al., 2017). It is logical to expect high variation amongst females, even in the same environment, due to fluctuating estrogen levels over time. While some past investigations could not find differences in gut microbiome tied to menstrual cycle phases (Krog et al., 2022) other have found large inter-individual differences in gut microbiome over time (Vandeputte et al., 2021) and consistent differences in composition based on sex, even with the same diet intervention (Dominianni et al., 2015).

Nonetheless, we attempted to produce a unique 3-feature model produced from the female mice, with the best possible R2M of only 0.15 coming from *Actinomycetales*, *Coriobacteriales* and *L. salivariu*s as predictors. There are known metabolic connections for all three of the features identified. Increased *Actinomycetales* has previously correlated with reduced obesity as seen in our model (Tarres et al., 2012). *Coriobacteriales* are higher in insulin-sensitive versus insulin resistant individuals (Yuan et al., 2021) and also correlate with lower inflammatory markers (Shahinozzaman et al., 2021) again corroborating our observations. *L. salivarius* is a well-studied species commonly added to probiotics due to their immunomodulatory activity and digestion of starch and amino acids, but their use has yielded mixed results (Chaves et al., 2017). Our experiments saw a decrease in their abundance, corresponding to experiments that saw increased weight with the addition of *L. salivarius* (Xia et al., 2021). However, other studies found supplementation prevented DIO (Liang et al., 2021), reduced stress-induced sugar craving (Nicol et al., 2023) and improved lipid metabolism (Chen et al., 2022). The true benefits of *L. salivarius* thus remains an open question.

Our female-specific 3-feature model performed exceptionally well in predicting weight change in the second female cohort (R=0.73, p<0.001) and explained a larger percentage of weight change variation at 32%. Interestingly, the male-produced 3-feature model had the best predictive value for the second female mouse cohort (R=0.89, p<0.001). And despite not accounting for much variation in weight change for the first female cohort (R2M 0.0063) the model still could accurately predict weight change (R=0.66, p<0.001). The 3-feature model’s predictive capacity demonstrates that variation in the specific bacterial predictors we identified are strongly correspond to weight status regardless of their level of abundance.

### 4.5 Study limitations and future directions

In our study, we did not measure behavioral alterations that potentially accompany THC supplementation, including changes in activity or food intake, that may significantly contribute to variation in weight loss. Manipulating the ECS not only directly alters brain behavior due to abundant CB1 receptors (Cuddihey et al., 2022), but there is also evidence microbiota can affect activity in regions like the hippocampus by altering eCB ligand levels (Chevalier et al., 2020) and modifying intestinal neural circuits that signal to the CNS (Obata et al., 2020). It would be prudent for subsequent experiments on ECS modulation to account for behavior and brain activity changes. Our investigation only tested one consistent THC dose of 10 mg/kg which corresponds to a 58mg dose in a 70 kg individual when scaled for surface area (Nair and Jacob, 2016). 58 mg is close to the median dose of edibles sampled from Oregon and Washington medical cannabis dispensaries and therefore closely models real-world consumption (Vandrey et al., 2015). Future research should analyze dose-dependent responses, especially since the effect of THC on appetite is known to shift from hyperphagia to hypophagia with increasing concentration (Bellocchio et al., 2010). Effects of ECS modulation can also be isolated utilizing mice with eCB receptor knockouts. Other trials may also consider examining how weight and microbiome responds after THC is halted; this approach is a first step to test a proposed hypothesis that eCB partial agonism leads to receptor downregulation (Le Foll et al., 2013). Additionally, we do not know which tissues decreased in mass during weight loss as adiposity was not measured. Finally, it is worth stating that we cannot infer THC effects on humans directly from our experiments and our results should not be misconstrued as advocating the use of cannabis to lose weight without extensive additional research.

Future investigations into interactions between the microbiome, ECS and metabolism must continue to account for sex differences. While there are known sex-dependent effects on the gut microbiome (Dominianni et al., 2015), a reciprocal modulatory axis between sex hormones like estrogen and microbiota composition (Baker et al., 2017) and differential effects on THC based on sex (Cooper and Craft, 2018), there remains many inconsistencies in findings on sex differences for the issues referenced (Cabal et al., 2018). Subsequent experiments should examine hormonal or other sex-reliant factors.

Our microbiome modeling was also limited by several elements. First, we cannot prove the features identified are causal of weight change without performing experiments on germ-free mice. To prove a causal effect of abundance on weight change, the specific bacteria should be directly introduced to mice without other microbial contamination. Furthermore, because genotype is a determinant of microbiota in addition to environment (Kovacs et al., 2011), our results cannot be extrapolated beyond the strain, species and population tested. Our significant bacterial features tended to come from the order or genus taxonomic levels. Achieving more specific findings in the genus or species may require larger sample sizes, continued advancement in taxonomic databases like Greengenes, and improvements in sequencing such as using the shotgun approach and longer reads. Consequently, we eliminated a number of taxonomies that could not be identified through QIIME2, potentially impacting our results. Current short-read next generation sequencing and 16S-based techniques may be inadequate to comprehensively capture important differences in composition as even microbes within the same species can produce diverse metabolites and thus interact with sex hormones or eCB differently (Kim et al., 2020). With regards to our mathematical strategy, while models utilize LME to account for variability within populations, the approach does come with its own set of limitations: LME still does not account for individual responses to fixed effects and requires its own set of complicated statistical interpretation (Brown, 2021). Of note, we did also attempt random forest analysis in R and QIIME2 to identify key taxonomic features to distinguish THC from VEH mice, to predict weight change, and to obtain values of feature importance all while limiting user bias in their selection (Strobol et al., 2007). However, several issues arose from the technique including over-fitting of data and selection of features that did not differ by treatment or lack significant correlation with weight change. Therefore, despite its limitations, we stand by the use of LME or similar modeling moving forward in identifying microbiome features of interest.

### 4.6 Conclusions

Our results demonstrate the weight loss potential of THC in DIO mice, thereby supporting ongoing efforts to investigate ECS regulation in combating obesity. We subsequently determined sex-specific gut microbiome changes related to THC administration and related to percent body mass change using a process of step-wise feature selection. Finally, we validated the concept of using advanced statistical modeling to generate predictions for health outcomes – such as variation in weight loss – with gut microbiome composition. Future experiments can expand upon our research to narrow down bacterial features that beneficially modulate ECS activity and lead to lowered body mass and adiposity. Based on recommendations by our lab for transparent microbiome data access (Kaye et al., 2024), our relative abundance measurements are readily available in the supplemental material.

## Data availability statement

The original contributions presented in the study are publicly available. This data can be found here: FigShare, doi: 10.6084/m9.figshare.26103214.v1.

## Ethics statement

The animal study was approved by Des Moines University Institutional Animal Care and Use Committee. The study was conducted in accordance with the local legislation and institutional requirements.

## Author contributions

AK: Formal analysis, Writing – original draft, Writing – review & editing. MR: Conceptualization, Formal analysis, Writing – review & editing. AD: Investigation, Writing – review & editing. PK: Investigation, Writing – review & editing. LW: Investigation, Writing – review & editing. KM: Conceptualization, Investigation, Methodology, Project administration, Writing – review & editing, Funding acquisition. LY: Conceptualization, Formal analysis, Funding acquisition, Methodology, Supervision, Writing – review & editing, Project administration.
