# Supplementary material for: Microbiome variations induced by delta9-tetrahydrocannabinol predict weight reduction in obese mice
Source: Front Microbiomes. 2024 Jul 16;3:1412468. doi: 10.3389/frmbi.2024.1412468 (PMC12993608; doi:10.3389/frmbi.2024.1412468)
Supplement: Supplementary file 1 [file DataSheet_1.pdf]

Frequency per feature

	Frequency
Minimum frequency	3.0
1st quartile	4.0
Median frequency	7.0
3rd quartile	38.0
Maximum frequency	1,488,120.0
Mean frequency	2,342.8513880192027

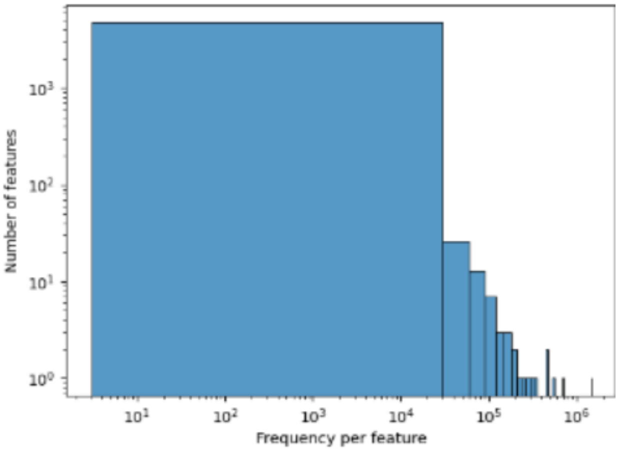

Table summary

Metric	Sample
Number of samples	135
Number of features	4,791
Total frequency	11,224,601

Frequency per sample

	Frequency
Minimum frequency	21,799.0
1st quartile	54,936.5
Median frequency	74,635.0
3rd quartile	107,479.5
Maximum frequency	208,575.0
Mean frequency	83,145.19259259259

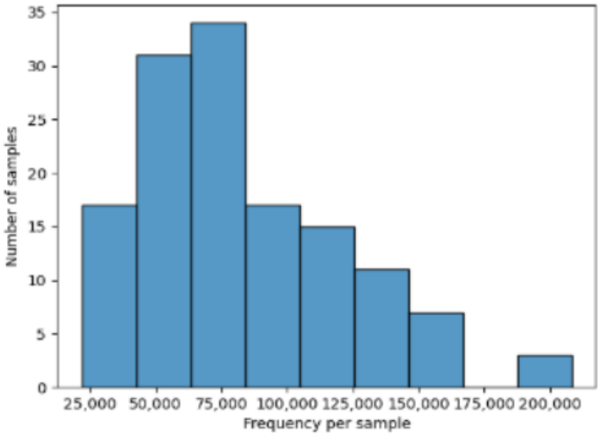

Supplementary Figure 1: Example QIIME2 Feature Table Output. This feature table was produced post DADA2 filtering for the original cohort (male and female mice). Data from this output were utilized to decide parameters for subsequent rarefaction to maximize samples included and to minimize insufficient sample.
