# Supplementary material for: Microbiome variations induced by delta9-tetrahydrocannabinol predict weight reduction in obese mice
Source: Front Microbiomes. 2024 Jul 16;3:1412468. doi: 10.3389/frmbi.2024.1412468 (PMC12993608; doi:10.3389/frmbi.2024.1412468)
Supplement: Supplementary file 2 [file DataSheet_2.pdf]

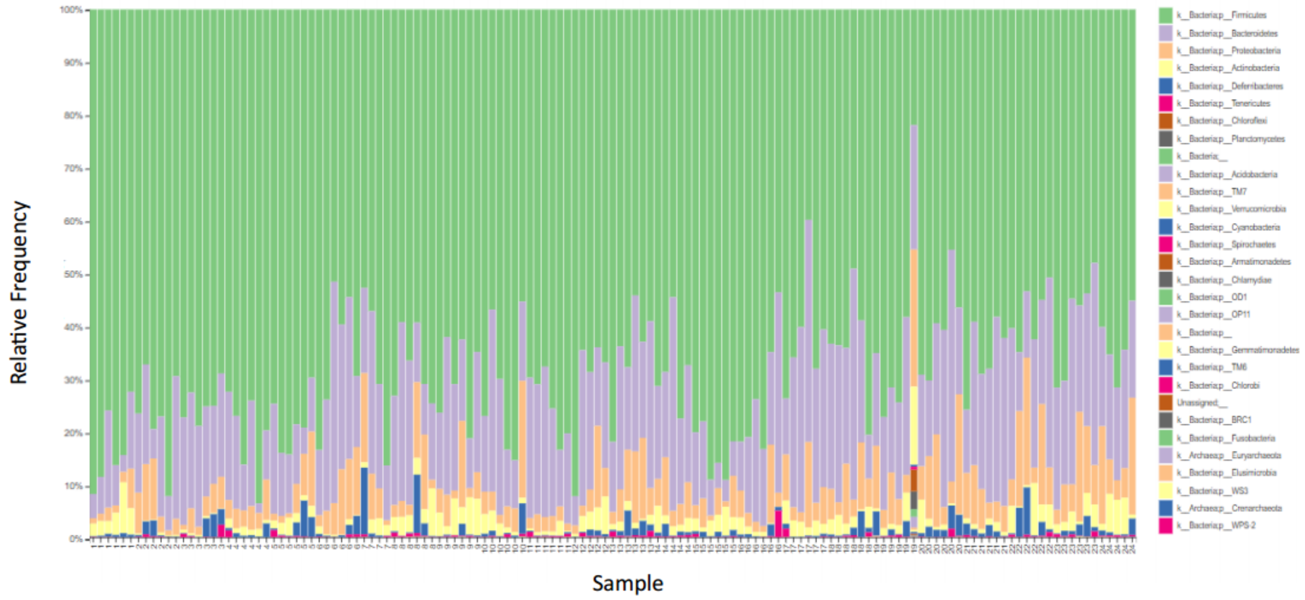

Supplementary Figure 2: Example QIIME2 Taxonomy Bar Plot. This bar plot is a visualization for the relative frequency of bacterial features in the first male mouse cohort at the second taxonomic level (phylum). Data export from QIIME2 taxonomic analysis provides an excel spreadsheet with absolute abundance that were manually converted to relative abundance for analysis. levels 2-7 (phylum to species) were all examined. Unnamed or unassigned features were not included in modeling.
