# Supplementary material for: Microbiome variations induced by delta9-tetrahydrocannabinol predict weight reduction in obese mice
Source: Front Microbiomes. 2024 Jul 16;3:1412468. doi: 10.3389/frmbi.2024.1412468 (PMC12993608; doi:10.3389/frmbi.2024.1412468)
Supplement: Supplementary file 3 [file DataSheet_3.pdf]

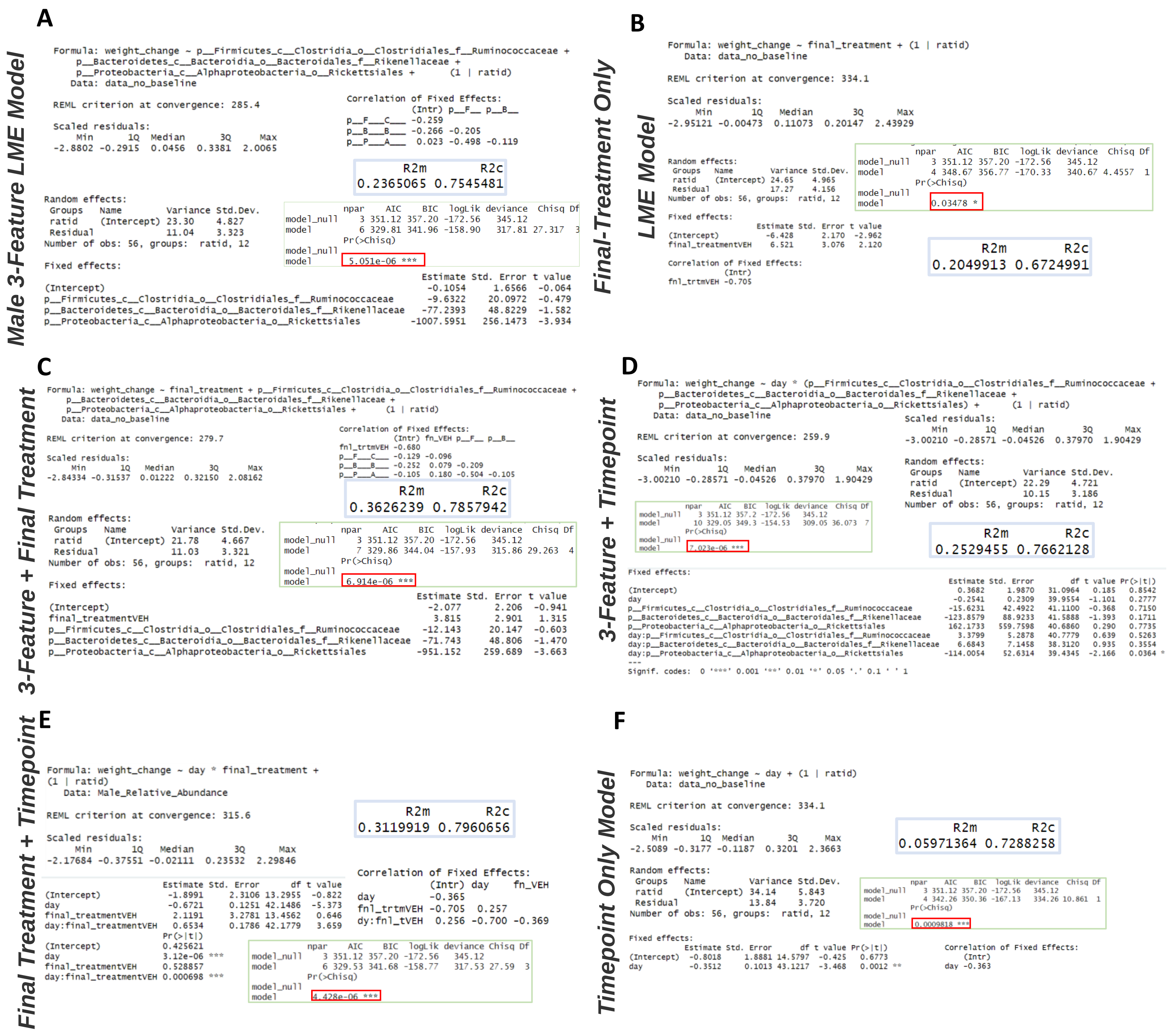

Supplementary Figure 3: R Summary Statistics for LME Percent Weight Change Models in Male Mice. LME models of percent weight change from baseline with different predictors. Each panel displays the results of summary(model) and of r.squaredGLMM(model) in R. Marginal R^2 (R2M) and conditional R^2 (R2C) are highlighted in the blue boxes. Results of likelihood ratio test against "null" model [lme(weight_change ~ 1+ (1|ratid))] are isolated in the green boxes with the p-value in red. **A)** Final LME "3-feature model" predicting percent weight change with relative abundance of 3 bacterial taxonomic features. **B)** LME model predicting weight change only using final treatment group. **C)** Addition of final treatment group identity to 3-feature model. **D)** Adding temporal effects (day) to 3-feature model. **E)** LME model of final treatment with temporal effects. **F)** LME model of temporal effects only.
