# Supplementary material for: Microbiome variations induced by delta9-tetrahydrocannabinol predict weight reduction in obese mice
Source: Front Microbiomes. 2024 Jul 16;3:1412468. doi: 10.3389/frmbi.2024.1412468 (PMC12993608; doi:10.3389/frmbi.2024.1412468)
Supplement: Supplementary file 4 [file DataSheet_4.pdf]

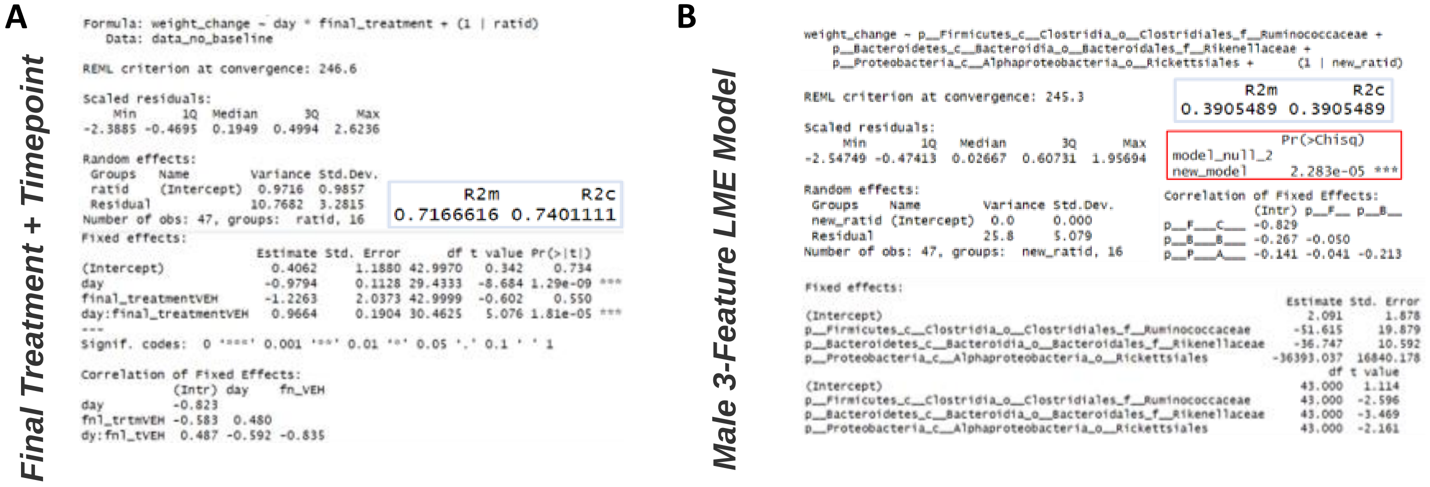

Supplementary Figure 4: R Summary Statistics Testing the Male-Produced 3-Feature LME Model on a Second Male Mouse Cohort. A) Results of summary(model) in R for an LME model of final treatment with temporal effects. **B)** Results of summary(model) in R for the 3-feature model in the second mouse cohort. Result of likelihood ratio test against null model is highlighted in the red box. Marginal R^2 (R2M) and conditional R^2 (R2C) are highlighted in the blue boxes.
