# Supplementary material for: Microbiome variations induced by delta9-tetrahydrocannabinol predict weight reduction in obese mice
Source: Front Microbiomes. 2024 Jul 16;3:1412468. doi: 10.3389/frmbi.2024.1412468 (PMC12993608; doi:10.3389/frmbi.2024.1412468)
Supplement: Supplementary file 5 [file DataSheet_5.pdf]

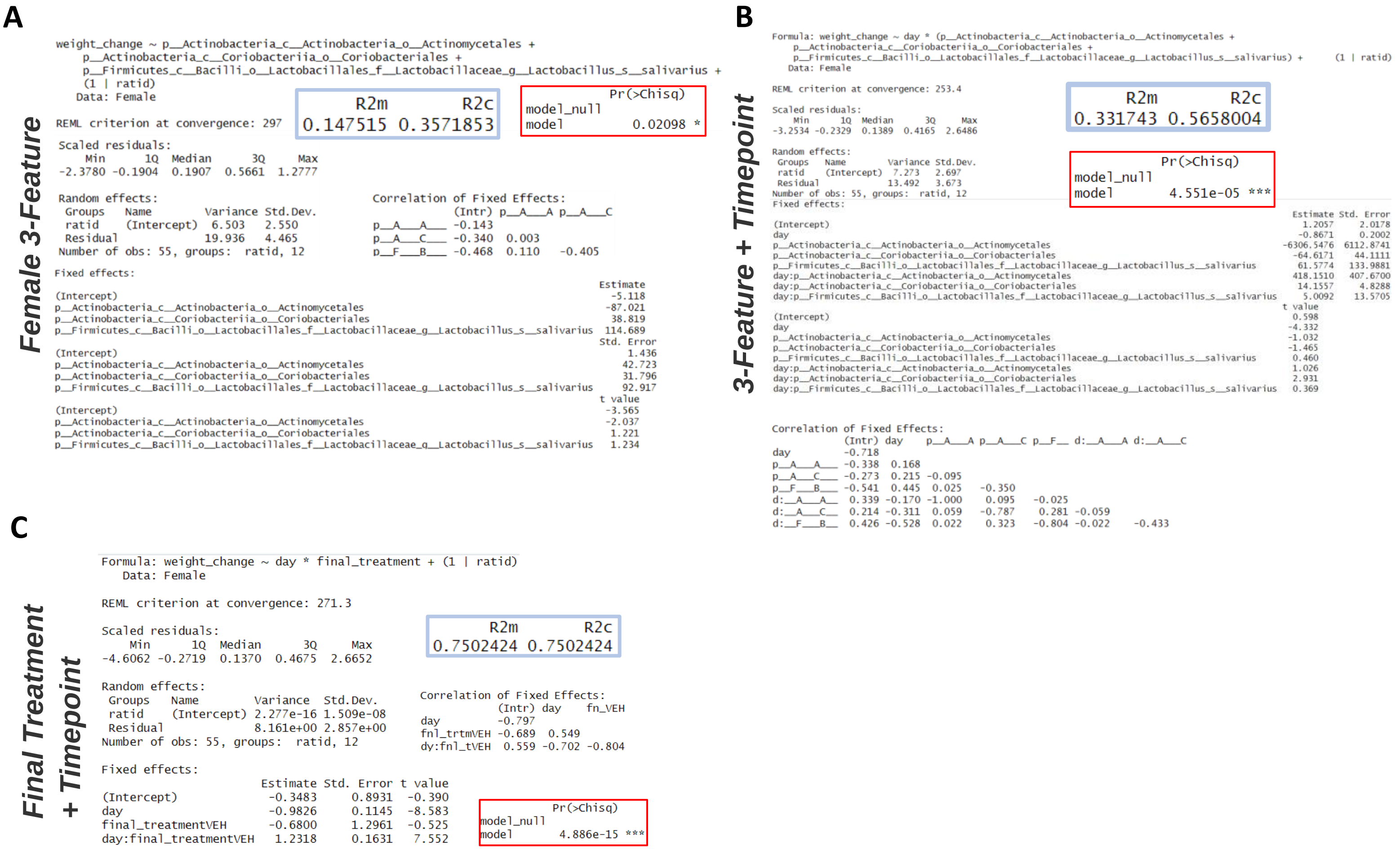

Supplementary Figure 5: R Summary Statistics of Taxonomic Analysis and LME Weight Change Modeling in Female Mice. LME models predicting percent weight change from baseline in female mice. Results of summary(model) and of r.squaredGLMM(model) in R. Marginal R^2 (R2M) and conditional R^2 (R2C) are highlighted in the blue box. Result of likelihood ratio test against null model is isolated in the red box. **A)** LME "3-feature model" for female mice predicting percent weight change using relative abundance of 3 bacterial taxonomic features. **B)** 3-Feature LME model for female mice with addition of timepoint (day). **C)** LME model for percent weight change using final treatment and timepoint (day).
