# Supplementary material for: Microbiome variations induced by delta9-tetrahydrocannabinol predict weight reduction in obese mice
Source: Front Microbiomes. 2024 Jul 16;3:1412468. doi: 10.3389/frmbi.2024.1412468 (PMC12993608; doi:10.3389/frmbi.2024.1412468)
Supplement: Supplementary file 6 [file DataSheet_6.pdf]

A

Cohort 2 Weight Analysis

```
Formula: weight_change ~ day * condition + (1 | new_ratid)
   Data: data_no_baseline_2

REML criterion at convergence: 166

Scaled residuals:
     Min       1Q   Median       3Q      Max
-2.43230 -0.37062  0.00488  0.44989  1.61281

Random effects:
 Groups    Name        Variance Std.Dev.
 new_ratid (Intercept) 10.17    3.190
 Residual              12.05    3.472
Number of obs: 30, groups:  new_ratid, 11

Fixed effects:
                 Estimate Std. Error t value
(Intercept)        1.9525     1.8490   1.056
day               -1.2228     0.1397  -8.756
conditionVEH      -3.7830     3.7689  -1.004
day:conditionVEH   1.2141     0.2862   4.242
```

```
      R2m       R2c
0.6576056 0.8143288
```

```
Correlation of Fixed Effects:
            (Intr) day    cndVEH
day         -0.682
conditinVEH -0.491  0.334
dy:cndtnVEH  0.333 -0.488 -0.723
```

B

Female 3-Feature on Cohort 2

```
Formula: weight_change ~ p__Actinobacteria + p__Actinobacteria_c__Coriobacteriia_o__Coriobacteriales +
    p__Firmicutes_c__Bacilli_o__Lactobacillales_f__Lactobacillaceae_g__Lactobacillus +      (1 | new_ratid)
   Data: data_no_baseline_2

REML criterion at convergence: 143.8

Scaled residuals:
     Min       1Q   Median       3Q      Max
-1.91850 -0.58403 -0.05095  0.66155  1.51589

Random effects:
 Groups    Name        Variance Std.Dev.
 new_ratid (Intercept) 11.49    3.389
 Residual              38.00    6.164
Number of obs: 30, groups:  new_ratid, 11
```

```
           Pr(>Chisq)
model_null
model       1.711e-05 ***
```

```
      R2m       R2c
0.3196729 0.4776002
```

```
Fixed effects:
                                                                            Estimate Std. Error t value
(Intercept)                                                                   -5.996      2.120  -2.828
p__Actinobacteria                                                          11703.733  57442.881   0.204
p__Actinobacteria_c__Coriobacteriia_o__Coriobacteriales                   -13734.435  57438.494  -0.239
p__Firmicutes_c__Bacilli_o__Lactobacillales_f__Lactobacillaceae_g__Lactobacillus   260.379    136.598   1.906

Correlation of Fixed Effects:
           (Intr) p__Act p__A__
p__Actnbctr -0.166
p__A___C___  0.160 -1.000
p__F___B___ -0.434 -0.183  0.184
```

C

Male 3-Feature on Female

```
Formula: weight_change ~ p__Firmicutes_c__Clostridia_o__Clostridiales_f__Ruminococcaceae +
    p__Bacteroidetes_c__Bacteroidia_o__Bacteroidales_f__Rikenellaceae +
    p__Proteobacteria_c__Alphaproteobacteria_o__Rickettsiales +      (1 | ratid)
   Data: data_no_baseline_2

REML criterion at convergence: 304.7

Scaled residuals:
    Min      1Q  Median      3Q     Max
-2.3647 -0.1419  0.1690  0.5632  1.3410

Random effects:
 Groups   Name        Variance Std.Dev.
 ratid    (Intercept) 10.87    3.297
 Residual             22.32    4.724
Number of obs: 55, groups:  ratid, 12
```

```
           Pr(>Chisq)
model_null
model         0.01585 *
```

```
        R2m       R2c
0.006264704 0.3317904
```

```
Correlation of Fixed Effects:
            (Intr) p__F__ p__B__
p__F___C___ -0.582
p__B___B___ -0.369 -0.251
p__P___A___  0.134 -0.317 -0.052
```

```
Fixed effects:
                                                                Estimate Std. Error     df t value Pr(>|t|)
(Intercept)                                                       -3.438      1.912 26.212  -1.798   0.0836 .
p__Firmicutes_c__Clostridia_o__Clostridiales_f__Ruminococcaceae    4.176     27.723 50.931   0.151   0.8809
p__Bacteroidetes_c__Bacteroidia_o__Bacteroidales_f__Rikenellaceae  7.577     46.181 50.765   0.164   0.8703
p__Proteobacteria_c__Alphaproteobacteria_o__Rickettsiales         81.183    179.692 46.889   0.452   0.6535
```

D

Male 3-Feature on Cohort 2 Females

```
Formula: weight_change ~ p__Bacteroidetes_c__Bacteroidia_o__Bacteroidales_f__Rikenellaceae +
    p__Firmicutes_c__Clostridia_o__Clostridiales_f__Ruminococcaceae +
    p__Proteobacteria_c__Alphaproteobacteria_o__Rickettsiales +      (1 | new_ratid)
   Data: data_no_baseline_2

REML criterion at convergence: 148.3

Scaled residuals:
     Min       1Q   Median       3Q      Max
-1.47168 -0.59545 -0.05896  0.51798  1.72772

Random effects:
 Groups    Name        Variance Std.Dev.
 new_ratid (Intercept) 32.46    5.697
 Residual              20.96    4.578
Number of obs: 30, groups:  new_ratid, 11
```

```
             Pr(>Chisq)
model_null_2
new_model     0.0004609 ***
```

```
      R2m       R2c
0.3724733 0.7538152
```

```
Correlation of Fixed Effects:
            (Intr) p__B__ p__F__
p__B___B___ -0.417
p__F___C___ -0.645 -0.133
p__P___A___ -0.100  0.192 -0.256
```

```
Fixed effects:
                                                                   Estimate Std. Error t value
(Intercept)                                                          10.062      3.563   2.824
p__Bacteroidetes_c__Bacteroidia_o__Bacteroidales_f__Rikenellaceae  -142.412     43.923  -3.242
p__Firmicutes_c__Clostridia_o__Clostridiales_f__Ruminococcaceae     -65.504     16.824  -3.893
p__Proteobacteria_c__Alphaproteobacteria_o__Rickettsiales        -43435.938  27303.651  -1.591
```

Supplementary Figure 6: R Summary Statistics Testing 3-Feature LME Models on a Second Female Mouse Cohort. LME models predicting percent weight change from baseline in female mice. Results of summary(model) and of r.squaredGLMM(model) in R. Marginal R^2 (R2M) and conditional R^2 (R2C) are highlighted in the blue box. Result of likelihood ratio test against null model is isolated in the red boxes. **A)** Testing the female-specific 3-feature LME model in the second cohort of female mice. **B)** Testing the male-produced 3-feature LME model in the original female mice cohort. **C)** Testing the male-produced 3-feature LME model in the second female mice cohort.
