# Supplementary material for: Microbiome variations induced by delta9-tetrahydrocannabinol predict weight reduction in obese mice
Source: Front Microbiomes. 2024 Jul 16;3:1412468. doi: 10.3389/frmbi.2024.1412468 (PMC12993608; doi:10.3389/frmbi.2024.1412468)
Supplement: Supplementary file 7 [file Table_1.docx]

| ratid | Day | Weight (g) | Weight Change (%) | Final Treatment |
| --- | --- | --- | --- | --- |
| 2 | 1 | 45.4 |  | THC |
| 2 | 2 | 44.9 | -1.10132 | THC |
| 2 | 3 | 43.5 | -4.18502 | THC |
| 2 | 4 | 41.6 | -8.37004 | THC |
| 2 | 9 | 36.5 | -19.6035 | THC |
| 2 | 15 | 35 | -22.9075 | THC |
| 3 | 1 | 51.4 |  | THC |
| 3 | 2 | 49.7 | -3.30739 | THC |
| 3 | 4 | 47.4 | -7.7821 | THC |
| 3 | 9 | 44.9 | -12.6459 | THC |
| 3 | 15 | 43.5 | -15.3696 | THC |
| 6 | 1 | 52 |  | THC |
| 6 | 2 | 48.9 | -5.96154 | THC |
| 6 | 3 | 47.4 | -13.9746 | THC |
| 6 | 4 | 46.5 | -15.608 | THC |
| 6 | 9 | 41.7 | -24.3194 | THC |
| 6 | 15 | 40 | -27.4047 | THC |
| 16 | 1 | 50.3 |  | THC |
| 16 | 2 | 50.1 | -0.00398 | THC |
| 16 | 3 | 49.5 | -0.0159 | THC |
| 16 | 4 | 48.2 | -0.04175 | THC |
| 16 | 9 | 44.8 | -0.10934 | THC |
| 16 | 15 | 43.3 | -0.13917 | THC |
| 17 | 2 | 52.9 | -0.00378 | THC |
| 17 | 1 | 52.7 |  | THC |
| 17 | 3 | 51.2 | -0.03214 | THC |
| 17 | 4 | 50.6 | -0.04348 | THC |
| 17 | 9 | 46 | -0.13043 | THC |
| 17 | 15 | 44.6 | -0.1569 | THC |
| 18 | 1 | 52.3 |  | THC |
| 18 | 2 | 52.1 | -0.00382 | THC |
| 18 | 3 | 51.6 | -0.01338 | THC |
| 18 | 4 | 50.2 | -0.04015 | THC |
| 18 | 9 | 45.7 | -0.1262 | THC |
| 18 | 15 | 44.1 | -0.15679 | THC |
| 1 | 1 | 55.3 |  | VEH |
| 1 | 2 | 55.4 | 0.180832 | VEH |
| 1 | 3 | 55.8 | 0.904159 | VEH |
| 1 | 4 | 55.6 | 0.542495 | VEH |
| 1 | 9 | 56 | 1.265823 | VEH |
| 1 | 15 | 56.3 | 1.808318 | VEH |
| 4 | 1 | 56 |  | VEH |
| 4 | 2 | 56.5 | 0.892857 | VEH |
| 4 | 3 | 55.9 | -0.17857 | VEH |
| 4 | 4 | 56.1 | 0.178571 | VEH |
| 4 | 9 | 55.2 | -1.42857 | VEH |
| 4 | 15 | 54 | -3.57143 | VEH |
| 5 | 1 | 54.3 |  | VEH |
| 5 | 2 | 54.2 | -0.18416 | VEH |
| 5 | 3 | 54.4 | 0.184162 | VEH |
| 5 | 9 | 54.5 | 0.368324 | VEH |
| 5 | 15 | 55.1 | 1.473297 | VEH |
| 13 | 1 | 52.8 |  | VEH |
| 13 | 2 | 52.7 | -0.00189 | VEH |
| 13 | 3 | 52.8 | 0 | VEH |
| 13 | 4 | 52.7 | -0.00189 | VEH |
| 13 | 15 | 52.4 | -0.00758 | VEH |
| 14 | 1 | 43.8 |  | VEH |
| 14 | 3 | 43.5 | -0.00685 | VEH |
| 14 | 4 | 43.1 | -0.01598 | VEH |
| 14 | 9 | 42.9 | -0.02055 | VEH |
| 14 | 15 | 43 | -0.01826 | VEH |
| 15 | 1 | 53 |  | VEH |
| 15 | 2 | 53 | 0 | VEH |
| 15 | 3 | 53.1 | 0.001887 | VEH |
| 15 | 4 | 53 | 0 | VEH |
| 15 | 9 | 52.7 | -0.00566 | VEH |
| 15 | 15 | 53.8 | 0.015094 | VEH |

**Supplementary Table 1: Weight Data for Male Mice.** Day 1 is the experimental baseline. Column “weight_change (%)” is the percent change in weight from baseline.
