# Supplementary material for: Microbiome variations induced by delta9-tetrahydrocannabinol predict weight reduction in obese mice
Source: Front Microbiomes. 2024 Jul 16;3:1412468. doi: 10.3389/frmbi.2024.1412468 (PMC12993608; doi:10.3389/frmbi.2024.1412468)
Supplement: Supplementary file 8 [file Table_2.docx]

| ratid | day | Weight (g) | Weight Change (%) | Final Treatment |
| --- | --- | --- | --- | --- |
| 7 | 1 | 34 |  | THC |
| 7 | 2 | 34.3 | 0.882353 | THC |
| 7 | 3 | 33.2 | -2.35294 | THC |
| 7 | 4 | 32.6 | -4.11765 | THC |
| 8 | 1 | 48 |  | VEH |
| 8 | 2 | 47.9 | -0.20833 | VEH |
| 8 | 3 | 47.8 | -0.41667 | VEH |
| 8 | 4 | 47.8 | -0.41667 | VEH |
| 8 | 9 | 48.8 | 1.666667 | VEH |
| 8 | 15 | 48.8 | 1.666667 | VEH |
| 9 | 1 | 37.6 |  | VEH |
| 9 | 2 | 37.9 | 0.797872 | VEH |
| 9 | 3 | 37.8 | 0.531915 | VEH |
| 9 | 4 | 37.7 | 0.265957 | VEH |
| 9 | 9 | 38.8 | 3.191489 | VEH |
| 9 | 15 | 40.3 | 7.180851 | VEH |
| 10 | 1 | 28.1 |  | THC |
| 10 | 2 | 27.2 | -3.20285 | THC |
| 10 | 3 | 26.5 | -5.69395 | THC |
| 10 | 4 | 23.2 | -17.4377 | THC |
| 10 | 9 | 25.8 | -8.18505 | THC |
| 10 | 15 | 26 | -7.47331 | THC |
| 11 | 1 | 35 |  | THC |
| 11 | 2 | 34.8 | -0.57143 | THC |
| 11 | 3 | 34 | -2.85714 | THC |
| 11 | 4 | 33 | -5.71429 | THC |
| 11 | 9 | 29.8 | -14.8571 | THC |
| 11 | 15 | 29.8 | -14.8571 | THC |
| 12 | 1 | 40.1 |  | VEH |
| 12 | 3 | 39.1 | -2.49377 | VEH |
| 12 | 4 | 38.9 | -2.99252 | VEH |
| 12 | 9 | 38.8 | -3.2419 | VEH |
| 19 | 1 | 44.2 |  | THC |
| 19 | 2 | 43.8 | -0.90498 | THC |
| 19 | 3 | 43.5 | -1.58371 | THC |
| 19 | 4 | 43 | -2.71493 | THC |
| 19 | 9 | 40.5 | -8.37104 | THC |
| 19 | 15 | 38 | -14.0271 | THC |
| 20 | 1 | 40 |  | THC |
| 20 | 2 | 39.7 | -0.75 | THC |
| 20 | 3 | 39.5 | -1.25 | THC |
| 20 | 4 | 39.2 | -2 | THC |
| 20 | 9 | 34.8 | -13 | THC |
| 20 | 15 | 33.3 | -16.75 | THC |
| 21 | 1 | 41.9 |  | THC |
| 21 | 2 | 41.6 | -0.71599 | THC |
| 21 | 3 | 41.2 | -1.67064 | THC |
| 21 | 4 | 40.6 | -3.10263 | THC |
| 21 | 9 | 37 | -11.6945 | THC |
| 21 | 15 | 35.3 | -15.7518 | THC |
| 22 | 1 | 40 |  | VEH |
| 22 | 2 | 39.7 | -0.75 | VEH |
| 22 | 3 | 39.9 | -0.25 | VEH |
| 22 | 4 | 40 | 0 | VEH |
| 22 | 9 | 39.9 | -0.25 | VEH |
| 22 | 15 | 40.1 | 0.25 | VEH |
| 23 | 1 | 39.1 |  | VEH |
| 23 | 2 | 39.4 | 0.767263 | VEH |
| 23 | 3 | 39.5 | 1.023018 | VEH |
| 23 | 4 | 39.4 | 0.767263 | VEH |
| 23 | 9 | 40.1 | 2.557545 | VEH |
| 23 | 15 | 40 | 2.30179 | VEH |
| 24 | 1 | 38.7 |  | VEH |
| 24 | 2 | 38.6 | -0.2584 | VEH |
| 24 | 3 | 38.4 | -0.77519 | VEH |
| 24 | 9 | 39.6 | 2.325581 | VEH |
| 24 | 15 | 39.9 | 3.100775 | VEH |

**Supplementary Table 2: Weight Data for Female Mice.** Day 1 is the experimental baseline. Column “weight_change (%)” is the percent change in weight from baseline.
