# Supplementary material for: Microbiome variations induced by delta9-tetrahydrocannabinol predict weight reduction in obese mice
Source: Front Microbiomes. 2024 Jul 16;3:1412468. doi: 10.3389/frmbi.2024.1412468 (PMC12993608; doi:10.3389/frmbi.2024.1412468)
Supplement: Supplementary file 9 [file Table_3.docx]

| ratid | day | final_treatment | *p__Firmicutes* | *p__Bacteroidetes* | *p__Proteobacteria* | *p__Actinobacteria* | *p__Deferribacteres* | *p__Tenericutes* | *c__Clostridia* | *c__Deferribacteres* | *c__Betaproteobacteria* | *c__Alphaproteobacteria* | *c__Epsilonproteobacteria* | *c__Mollicutes* | *o__Desulfovibrionales* | *o__Deferribacterales* | *o__Burkholderiales* | *o__Campylobacterales* | *o__Mycoplasmatales* |
| --- | --- | --- | --- | --- | --- | --- | --- | --- | --- | --- | --- | --- | --- | --- | --- | --- | --- | --- | --- |
| 2 | 1 | THC | 0.7715 | 0.1893 | 0.0307 | 0.0052 | 0.0022 | 0.0011 | 0.0917 | 0.0022 | 0.0028 | 0.0003 | 0.0024 | 0.0011 | 0.0252 | 0.0022 | 0.0028 | 0.0024 | 0.0011 |
| 2 | 2 | THC | 0.9222 | 0.0646 | 0.0095 | 0.0024 | 0.0007 | 0.0002 | 0.0370 | 0.0007 | 0.0041 | 0.0000 | 0.0014 | 0.0002 | 0.0036 | 0.0007 | 0.0041 | 0.0014 | 0.0002 |
| 2 | 3 | THC | 0.6950 | 0.2698 | 0.0312 | 0.0029 | 0.0009 | 0.0000 | 0.0989 | 0.0009 | 0.0146 | 0.0006 | 0.0006 | 0.0000 | 0.0150 | 0.0009 | 0.0146 | 0.0006 | 0.0000 |
| 2 | 4 | THC | 0.7657 | 0.1497 | 0.0779 | 0.0028 | 0.0031 | 0.0008 | 0.1190 | 0.0031 | 0.0141 | 0.0017 | 0.0033 | 0.0008 | 0.0588 | 0.0031 | 0.0141 | 0.0033 | 0.0008 |
| 2 | 9 | THC | 0.6726 | 0.1887 | 0.1075 | 0.0013 | 0.0245 | 0.0052 | 0.2776 | 0.0245 | 0.0000 | 0.0054 | 0.0114 | 0.0052 | 0.0905 | 0.0245 | 0.0000 | 0.0114 | 0.0052 |
| 2 | 15 | THC | 0.7951 | 0.0556 | 0.1162 | 0.0013 | 0.0299 | 0.0016 | 0.2474 | 0.0299 | 0.0002 | 0.0050 | 0.0145 | 0.0016 | 0.0966 | 0.0299 | 0.0002 | 0.0145 | 0.0016 |
| 3 | 1 | THC | 0.7730 | 0.2036 | 0.0094 | 0.0060 | 0.0016 | 0.0063 | 0.0343 | 0.0016 | 0.0033 | 0.0003 | 0.0010 | 0.0063 | 0.0036 | 0.0016 | 0.0033 | 0.0010 | 0.0063 |
| 3 | 2 | THC | 0.7260 | 0.2190 | 0.0479 | 0.0043 | 0.0011 | 0.0015 | 0.1609 | 0.0011 | 0.0155 | 0.0006 | 0.0085 | 0.0015 | 0.0233 | 0.0011 | 0.0155 | 0.0085 | 0.0015 |
| 3 | 4 | THC | 0.7524 | 0.1704 | 0.0373 | 0.0043 | 0.0346 | 0.0000 | 0.0933 | 0.0346 | 0.0193 | 0.0082 | 0.0036 | 0.0000 | 0.0054 | 0.0346 | 0.0193 | 0.0036 | 0.0000 |
| 3 | 9 | THC | 0.7511 | 0.1477 | 0.0552 | 0.0022 | 0.0431 | 0.0003 | 0.1409 | 0.0431 | 0.0010 | 0.0068 | 0.0052 | 0.0003 | 0.0416 | 0.0431 | 0.0010 | 0.0052 | 0.0003 |
| 3 | 15 | THC | 0.6898 | 0.1955 | 0.0604 | 0.0011 | 0.0296 | 0.0232 | 0.1860 | 0.0296 | 0.0001 | 0.0049 | 0.0011 | 0.0232 | 0.0541 | 0.0296 | 0.0001 | 0.0011 | 0.0232 |
| 6 | 1 | THC | 0.8348 | 0.1232 | 0.0219 | 0.0139 | 0.0051 | 0.0011 | 0.2839 | 0.0051 | 0.0002 | 0.0000 | 0.0030 | 0.0011 | 0.0185 | 0.0051 | 0.0002 | 0.0030 | 0.0011 |
| 6 | 2 | THC | 0.7394 | 0.2102 | 0.0449 | 0.0028 | 0.0020 | 0.0004 | 0.1911 | 0.0020 | 0.0093 | 0.0007 | 0.0052 | 0.0004 | 0.0291 | 0.0020 | 0.0093 | 0.0052 | 0.0004 |
| 6 | 3 | THC | 0.5159 | 0.4194 | 0.0604 | 0.0032 | 0.0008 | 0.0002 | 0.3294 | 0.0008 | 0.0073 | 0.0012 | 0.0081 | 0.0002 | 0.0434 | 0.0008 | 0.0073 | 0.0081 | 0.0002 |
| 6 | 4 | THC | 0.5970 | 0.2739 | 0.1246 | 0.0009 | 0.0033 | 0.0000 | 0.5277 | 0.0033 | 0.0018 | 0.0019 | 0.0332 | 0.0000 | 0.0869 | 0.0033 | 0.0018 | 0.0332 | 0.0000 |
| 6 | 9 | THC | 0.5453 | 0.3063 | 0.1143 | 0.0109 | 0.0180 | 0.0051 | 0.1819 | 0.0180 | 0.0137 | 0.0021 | 0.0149 | 0.0051 | 0.0836 | 0.0180 | 0.0136 | 0.0149 | 0.0051 |
| 6 | 15 | THC | 0.6947 | 0.1343 | 0.1292 | 0.0014 | 0.0350 | 0.0049 | 0.3436 | 0.0350 | 0.0005 | 0.0126 | 0.0018 | 0.0049 | 0.1134 | 0.0350 | 0.0005 | 0.0018 | 0.0049 |
| 16 | 1 | THC | 0.8191 | 0.0925 | 0.0514 | 0.0321 | 0.0045 | 0.0004 | 0.0950 | 0.0045 | 0.0183 | 0.0000 | 0.0095 | 0.0004 | 0.0236 | 0.0045 | 0.0183 | 0.0095 | 0.0004 |
| 16 | 2 | THC | 0.8103 | 0.1435 | 0.0264 | 0.0142 | 0.0055 | 0.0000 | 0.0822 | 0.0055 | 0.0004 | 0.0002 | 0.0078 | 0.0000 | 0.0178 | 0.0055 | 0.0004 | 0.0078 | 0.0000 |
| 16 | 3 | THC | 0.7384 | 0.2323 | 0.0191 | 0.0087 | 0.0015 | 0.0001 | 0.1503 | 0.0015 | 0.0034 | 0.0017 | 0.0043 | 0.0001 | 0.0097 | 0.0015 | 0.0034 | 0.0043 | 0.0001 |
| 16 | 4 | THC | 0.8330 | 0.1456 | 0.0117 | 0.0086 | 0.0003 | 0.0004 | 0.1329 | 0.0003 | 0.0017 | 0.0014 | 0.0019 | 0.0005 | 0.0062 | 0.0003 | 0.0017 | 0.0019 | 0.0005 |
| 16 | 9 | THC | 0.6492 | 0.1755 | 0.1490 | 0.0021 | 0.0222 | 0.0015 | 0.4581 | 0.0222 | 0.0003 | 0.0016 | 0.0053 | 0.0015 | 0.1415 | 0.0222 | 0.0003 | 0.0053 | 0.0014 |
| 16 | 15 | THC | 0.5362 | 0.3794 | 0.0267 | 0.0004 | 0.0070 | 0.0502 | 0.2763 | 0.0070 | 0.0002 | 0.0004 | 0.0039 | 0.0502 | 0.0223 | 0.0070 | 0.0002 | 0.0039 | 0.0502 |
| 17 | 2 | THC | 0.6809 | 0.2537 | 0.0344 | 0.0287 | 0.0023 | 0.0000 | 0.0672 | 0.0023 | 0.0222 | 0.0000 | 0.0037 | 0.0000 | 0.0085 | 0.0023 | 0.0222 | 0.0037 | 0.0000 |
| 17 | 1 | THC | 0.6065 | 0.2986 | 0.0696 | 0.0189 | 0.0048 | 0.0013 | 0.1077 | 0.0048 | 0.0151 | 0.0000 | 0.0044 | 0.0013 | 0.0499 | 0.0048 | 0.0151 | 0.0044 | 0.0013 |
| 17 | 3 | THC | 0.6593 | 0.2837 | 0.0252 | 0.0310 | 0.0009 | 0.0000 | 0.0590 | 0.0009 | 0.0177 | 0.0000 | 0.0010 | 0.0000 | 0.0065 | 0.0009 | 0.0177 | 0.0010 | 0.0000 |
| 17 | 4 | THC | 0.6020 | 0.3507 | 0.0185 | 0.0280 | 0.0008 | 0.0000 | 0.0432 | 0.0008 | 0.0113 | 0.0000 | 0.0025 | 0.0000 | 0.0047 | 0.0008 | 0.0113 | 0.0025 | 0.0000 |
| 17 | 9 | THC | 0.3987 | 0.4206 | 0.1621 | 0.0157 | 0.0022 | 0.0005 | 0.2552 | 0.0022 | 0.0012 | 0.0011 | 0.0092 | 0.0005 | 0.1505 | 0.0022 | 0.0012 | 0.0092 | 0.0001 |
| 17 | 15 | THC | 0.7372 | 0.1057 | 0.0878 | 0.0439 | 0.0092 | 0.0156 | 0.2527 | 0.0092 | 0.0007 | 0.0033 | 0.0028 | 0.0156 | 0.0810 | 0.0092 | 0.0007 | 0.0028 | 0.0150 |
| 18 | 1 | THC | 0.6334 | 0.2749 | 0.0579 | 0.0289 | 0.0042 | 0.0007 | 0.1027 | 0.0042 | 0.0107 | 0.0000 | 0.0036 | 0.0007 | 0.0434 | 0.0042 | 0.0107 | 0.0036 | 0.0007 |
| 18 | 2 | THC | 0.6365 | 0.2973 | 0.0244 | 0.0393 | 0.0021 | 0.0004 | 0.0444 | 0.0021 | 0.0105 | 0.0000 | 0.0078 | 0.0004 | 0.0061 | 0.0021 | 0.0105 | 0.0078 | 0.0002 |
| 18 | 3 | THC | 0.6414 | 0.2190 | 0.1140 | 0.0204 | 0.0027 | 0.0022 | 0.2669 | 0.0027 | 0.0319 | 0.0032 | 0.0127 | 0.0022 | 0.0661 | 0.0027 | 0.0319 | 0.0127 | 0.0014 |
| 18 | 4 | THC | 0.4913 | 0.4373 | 0.0324 | 0.0209 | 0.0144 | 0.0034 | 0.1497 | 0.0144 | 0.0153 | 0.0046 | 0.0062 | 0.0034 | 0.0064 | 0.0144 | 0.0153 | 0.0062 | 0.0027 |
| 18 | 9 | THC | 0.5895 | 0.2310 | 0.1273 | 0.0032 | 0.0488 | 0.0002 | 0.5353 | 0.0488 | 0.0001 | 0.0095 | 0.0181 | 0.0002 | 0.0996 | 0.0488 | 0.0001 | 0.0181 | 0.0000 |
| 18 | 15 | THC | 0.8061 | 0.0826 | 0.0538 | 0.0400 | 0.0086 | 0.0078 | 0.3055 | 0.0086 | 0.0005 | 0.0024 | 0.0027 | 0.0078 | 0.0481 | 0.0086 | 0.0005 | 0.0027 | 0.0065 |
| 1 | 1 | VEH | 0.9181 | 0.0458 | 0.0103 | 0.0240 | 0.0012 | 0.0006 | 0.0948 | 0.0012 | 0.0005 | 0.0002 | 0.0005 | 0.0006 | 0.0091 | 0.0012 | 0.0005 | 0.0005 | 0.0006 |
| 1 | 2 | VEH | 0.8857 | 0.0697 | 0.0142 | 0.0278 | 0.0023 | 0.0003 | 0.0561 | 0.0023 | 0.0027 | 0.0002 | 0.0015 | 0.0003 | 0.0096 | 0.0023 | 0.0027 | 0.0015 | 0.0003 |
| 1 | 3 | VEH | 0.7600 | 0.1835 | 0.0261 | 0.0241 | 0.0053 | 0.0006 | 0.0768 | 0.0053 | 0.0051 | 0.0000 | 0.0032 | 0.0006 | 0.0178 | 0.0053 | 0.0051 | 0.0032 | 0.0006 |
| 1 | 4 | VEH | 0.8636 | 0.0762 | 0.0141 | 0.0418 | 0.0040 | 0.0002 | 0.0773 | 0.0040 | 0.0010 | 0.0003 | 0.0028 | 0.0002 | 0.0101 | 0.0040 | 0.0010 | 0.0028 | 0.0002 |
| 1 | 9 | VEH | 0.8444 | 0.0314 | 0.0202 | 0.0964 | 0.0075 | 0.0001 | 0.3463 | 0.0075 | 0.0008 | 0.0000 | 0.0012 | 0.0001 | 0.0182 | 0.0075 | 0.0008 | 0.0012 | 0.0000 |
| 1 | 15 | VEH | 0.7245 | 0.1453 | 0.0754 | 0.0501 | 0.0045 | 0.0001 | 0.1197 | 0.0045 | 0.0033 | 0.0001 | 0.0054 | 0.0001 | 0.0666 | 0.0045 | 0.0032 | 0.0054 | 0.0001 |
| 4 | 1 | VEH | 0.7245 | 0.2049 | 0.0359 | 0.0175 | 0.0045 | 0.0127 | 0.0968 | 0.0045 | 0.0001 | 0.0001 | 0.0016 | 0.0127 | 0.0332 | 0.0045 | 0.0001 | 0.0016 | 0.0127 |
| 4 | 2 | VEH | 0.7702 | 0.1758 | 0.0373 | 0.0093 | 0.0074 | 0.0000 | 0.1253 | 0.0074 | 0.0040 | 0.0000 | 0.0023 | 0.0000 | 0.0310 | 0.0074 | 0.0040 | 0.0023 | 0.0000 |
| 4 | 3 | VEH | 0.8625 | 0.0865 | 0.0314 | 0.0169 | 0.0025 | 0.0000 | 0.1198 | 0.0025 | 0.0096 | 0.0000 | 0.0039 | 0.0000 | 0.0173 | 0.0025 | 0.0096 | 0.0039 | 0.0000 |
| 4 | 4 | VEH | 0.7414 | 0.1994 | 0.0454 | 0.0101 | 0.0037 | 0.0000 | 0.1136 | 0.0037 | 0.0017 | 0.0000 | 0.0030 | 0.0000 | 0.0407 | 0.0037 | 0.0017 | 0.0030 | 0.0000 |
| 4 | 9 | VEH | 0.9358 | 0.0180 | 0.0309 | 0.0142 | 0.0012 | 0.0000 | 0.2101 | 0.0012 | 0.0019 | 0.0000 | 0.0008 | 0.0000 | 0.0282 | 0.0012 | 0.0019 | 0.0008 | 0.0000 |
| 4 | 15 | VEH | 0.7977 | 0.0928 | 0.0769 | 0.0065 | 0.0258 | 0.0000 | 0.2198 | 0.0258 | 0.0037 | 0.0000 | 0.0193 | 0.0000 | 0.0539 | 0.0258 | 0.0037 | 0.0193 | 0.0000 |
| 5 | 1 | VEH | 0.7472 | 0.2082 | 0.0132 | 0.0148 | 0.0031 | 0.0132 | 0.0719 | 0.0031 | 0.0001 | 0.0001 | 0.0013 | 0.0132 | 0.0116 | 0.0031 | 0.0001 | 0.0013 | 0.0132 |
| 5 | 2 | VEH | 0.8403 | 0.1193 | 0.0133 | 0.0226 | 0.0018 | 0.0026 | 0.0815 | 0.0018 | 0.0001 | 0.0001 | 0.0005 | 0.0026 | 0.0127 | 0.0018 | 0.0001 | 0.0005 | 0.0025 |
| 5 | 3 | VEH | 0.8434 | 0.1110 | 0.0087 | 0.0343 | 0.0015 | 0.0010 | 0.1534 | 0.0015 | 0.0001 | 0.0001 | 0.0006 | 0.0010 | 0.0079 | 0.0015 | 0.0001 | 0.0006 | 0.0010 |
| 5 | 9 | VEH | 0.7922 | 0.0498 | 0.0787 | 0.0099 | 0.0679 | 0.0012 | 0.3665 | 0.0679 | 0.0000 | 0.0003 | 0.0356 | 0.0012 | 0.0427 | 0.0679 | 0.0000 | 0.0356 | 0.0012 |
| 5 | 15 | VEH | 0.6978 | 0.1018 | 0.1413 | 0.0210 | 0.0374 | 0.0005 | 0.2057 | 0.0374 | 0.0003 | 0.0001 | 0.0878 | 0.0005 | 0.0530 | 0.0374 | 0.0003 | 0.0878 | 0.0005 |
| 13 | 1 | VEH | 0.8195 | 0.1323 | 0.0239 | 0.0105 | 0.0032 | 0.0101 | 0.0445 | 0.0032 | 0.0013 | 0.0008 | 0.0019 | 0.0101 | 0.0199 | 0.0032 | 0.0013 | 0.0019 | 0.0092 |
| 13 | 2 | VEH | 0.6382 | 0.2713 | 0.0611 | 0.0181 | 0.0088 | 0.0024 | 0.1215 | 0.0088 | 0.0118 | 0.0014 | 0.0147 | 0.0024 | 0.0328 | 0.0088 | 0.0118 | 0.0147 | 0.0015 |
| 13 | 3 | VEH | 0.6780 | 0.1560 | 0.0977 | 0.0179 | 0.0483 | 0.0008 | 0.0897 | 0.0483 | 0.0139 | 0.0028 | 0.0034 | 0.0008 | 0.0773 | 0.0483 | 0.0139 | 0.0034 | 0.0003 |
| 13 | 4 | VEH | 0.5420 | 0.2953 | 0.1354 | 0.0108 | 0.0129 | 0.0032 | 0.1432 | 0.0129 | 0.0020 | 0.0004 | 0.0044 | 0.0032 | 0.1281 | 0.0129 | 0.0020 | 0.0044 | 0.0012 |
| 13 | 15 | VEH | 0.5909 | 0.3157 | 0.0510 | 0.0186 | 0.0122 | 0.0114 | 0.0965 | 0.0122 | 0.0016 | 0.0002 | 0.0089 | 0.0114 | 0.0403 | 0.0122 | 0.0016 | 0.0089 | 0.0101 |
| 14 | 1 | VEH | 0.7135 | 0.1757 | 0.0517 | 0.0506 | 0.0067 | 0.0018 | 0.1659 | 0.0067 | 0.0030 | 0.0000 | 0.0049 | 0.0018 | 0.0423 | 0.0067 | 0.0027 | 0.0049 | 0.0018 |
| 14 | 3 | VEH | 0.6871 | 0.1612 | 0.1106 | 0.0160 | 0.0239 | 0.0010 | 0.3393 | 0.0239 | 0.0015 | 0.0011 | 0.0165 | 0.0010 | 0.0912 | 0.0239 | 0.0014 | 0.0165 | 0.0007 |
| 14 | 4 | VEH | 0.5454 | 0.4043 | 0.0299 | 0.0177 | 0.0014 | 0.0014 | 0.1243 | 0.0014 | 0.0120 | 0.0001 | 0.0042 | 0.0014 | 0.0120 | 0.0014 | 0.0120 | 0.0042 | 0.0014 |
| 14 | 9 | VEH | 0.7752 | 0.1617 | 0.0396 | 0.0130 | 0.0074 | 0.0028 | 0.3581 | 0.0074 | 0.0002 | 0.0009 | 0.0016 | 0.0028 | 0.0366 | 0.0074 | 0.0002 | 0.0016 | 0.0027 |
| 14 | 15 | VEH | 0.6743 | 0.2212 | 0.0680 | 0.0271 | 0.0053 | 0.0039 | 0.1750 | 0.0053 | 0.0006 | 0.0012 | 0.0049 | 0.0039 | 0.0614 | 0.0053 | 0.0006 | 0.0049 | 0.0039 |
| 15 | 1 | VEH | 0.8012 | 0.1379 | 0.0260 | 0.0237 | 0.0051 | 0.0056 | 0.0688 | 0.0051 | 0.0012 | 0.0000 | 0.0009 | 0.0056 | 0.0239 | 0.0051 | 0.0012 | 0.0009 | 0.0040 |
| 15 | 2 | VEH | 0.7807 | 0.1454 | 0.0554 | 0.0085 | 0.0098 | 0.0000 | 0.5074 | 0.0098 | 0.0008 | 0.0019 | 0.0074 | 0.0000 | 0.0445 | 0.0098 | 0.0008 | 0.0074 | 0.0000 |
| 15 | 3 | VEH | 0.8911 | 0.0674 | 0.0098 | 0.0299 | 0.0015 | 0.0002 | 0.1607 | 0.0015 | 0.0004 | 0.0001 | 0.0022 | 0.0002 | 0.0065 | 0.0015 | 0.0004 | 0.0022 | 0.0002 |
| 15 | 4 | VEH | 0.8592 | 0.0477 | 0.0505 | 0.0325 | 0.0095 | 0.0005 | 0.1481 | 0.0095 | 0.0006 | 0.0010 | 0.0019 | 0.0006 | 0.0470 | 0.0095 | 0.0006 | 0.0019 | 0.0006 |
| 15 | 9 | VEH | 0.8916 | 0.0402 | 0.0105 | 0.0550 | 0.0022 | 0.0005 | 0.0781 | 0.0022 | 0.0010 | 0.0001 | 0.0010 | 0.0005 | 0.0078 | 0.0022 | 0.0009 | 0.0010 | 0.0005 |
| 15 | 15 | VEH | 0.8183 | 0.0653 | 0.0784 | 0.0245 | 0.0116 | 0.0017 | 0.1376 | 0.0116 | 0.0009 | 0.0008 | 0.0031 | 0.0017 | 0.0732 | 0.0116 | 0.0009 | 0.0031 | 0.0017 |

| *o__Rickettsiales* | *f__Ruminococcaceae* | *f__Desulfovibrionaceae* | *f__Lactobacillaceae* | *f__Bacteroidaceae* | *f__Rikenellaceae* | *f__Deferribacteraceae* | *f__Odoribacteraceae* | *f__Peptococcaceae* | *f__Mycoplasmataceae* | *f__Prevotellaceae* | *g__Ruminococcus* | *g__Bacteroides* | *g__Ruminococcus* | *g__Enterococcus* | *g__Desulfovibrio* | *g__Prevotella* | *s__indistinctus* | *s__salivarius* |
| --- | --- | --- | --- | --- | --- | --- | --- | --- | --- | --- | --- | --- | --- | --- | --- | --- | --- | --- |
| 0.0003 | 0.0180 | 0.0252 | 0.0323 | 0.0833 | 0.0097 | 0.0022 | 0.0131 | 0.0008 | 0.0011 | 0.0000 | 0.0075 | 0.1823 | 0.0331 | 0.0002 | 0.0007 | 0.0000 | 0.0003 | 0.0017 |
| 0.0000 | 0.0076 | 0.0036 | 0.0630 | 0.0324 | 0.0012 | 0.0007 | 0.0021 | 0.0004 | 0.0002 | 0.0000 | 0.0018 | 0.0345 | 0.0061 | 0.0001 | 0.0001 | 0.0000 | 0.0001 | 0.0030 |
| 0.0005 | 0.0088 | 0.0150 | 0.0494 | 0.1761 | 0.0039 | 0.0009 | 0.0125 | 0.0006 | 0.0000 | 0.0000 | 0.0012 | 0.1476 | 0.0131 | 0.0004 | 0.0002 | 0.0000 | 0.0007 | 0.0125 |
| 0.0017 | 0.0218 | 0.0588 | 0.0094 | 0.0503 | 0.0150 | 0.0031 | 0.0389 | 0.0009 | 0.0008 | 0.0000 | 0.0061 | 0.0503 | 0.0139 | 0.0000 | 0.0001 | 0.0000 | 0.0000 | 0.0034 |
| 0.0054 | 0.0724 | 0.0905 | 0.0009 | 0.0646 | 0.0289 | 0.0245 | 0.0331 | 0.0038 | 0.0052 | 0.0001 | 0.0227 | 0.0646 | 0.0110 | 0.0000 | 0.0013 | 0.0001 | 0.0002 | 0.0009 |
| 0.0050 | 0.0648 | 0.0966 | 0.0145 | 0.0136 | 0.0129 | 0.0299 | 0.0102 | 0.0015 | 0.0016 | 0.0005 | 0.0228 | 0.0136 | 0.0201 | 0.0000 | 0.0015 | 0.0005 | 0.0000 | 0.0035 |
| 0.0003 | 0.0057 | 0.0036 | 0.0608 | 0.0858 | 0.0115 | 0.0016 | 0.0116 | 0.0002 | 0.0063 | 0.0001 | 0.0017 | 0.0858 | 0.0059 | 0.0002 | 0.0002 | 0.0001 | 0.0000 | 0.0047 |
| 0.0006 | 0.0218 | 0.0233 | 0.1261 | 0.1235 | 0.0058 | 0.0011 | 0.0043 | 0.0002 | 0.0015 | 0.0001 | 0.0040 | 0.1235 | 0.0318 | 0.0000 | 0.0004 | 0.0001 | 0.0001 | 0.0215 |
| 0.0082 | 0.0147 | 0.0054 | 0.0166 | 0.0875 | 0.0113 | 0.0346 | 0.0333 | 0.0000 | 0.0000 | 0.0000 | 0.0114 | 0.0700 | 0.0008 | 0.0000 | 0.0001 | 0.0000 | 0.0000 | 0.0029 |
| 0.0068 | 0.0376 | 0.0416 | 0.0052 | 0.0690 | 0.0165 | 0.0431 | 0.0195 | 0.0010 | 0.0003 | 0.0000 | 0.0135 | 0.0499 | 0.0151 | 0.0000 | 0.0008 | 0.0000 | 0.0000 | 0.0092 |
| 0.0049 | 0.0483 | 0.0541 | 0.0897 | 0.0153 | 0.0327 | 0.0296 | 0.0719 | 0.0007 | 0.0232 | 0.0003 | 0.0224 | 0.0194 | 0.0245 | 0.0003 | 0.0011 | 0.0003 | 0.0002 | 0.0067 |
| 0.0000 | 0.0517 | 0.0185 | 0.0165 | 0.0407 | 0.0152 | 0.0051 | 0.0102 | 0.0011 | 0.0011 | 0.0000 | 0.0071 | 0.0601 | 0.0888 | 0.0000 | 0.0003 | 0.0000 | 0.0002 | 0.0160 |
| 0.0007 | 0.0318 | 0.0291 | 0.1443 | 0.1108 | 0.0121 | 0.0020 | 0.0174 | 0.0001 | 0.0004 | 0.0000 | 0.0102 | 0.1217 | 0.0449 | 0.0226 | 0.0008 | 0.0000 | 0.0004 | 0.0348 |
| 0.0012 | 0.0419 | 0.0434 | 0.0373 | 0.2246 | 0.0159 | 0.0008 | 0.0305 | 0.0024 | 0.0002 | 0.0000 | 0.0023 | 0.1120 | 0.0252 | 0.0020 | 0.0004 | 0.0000 | 0.0000 | 0.0077 |
| 0.0018 | 0.0630 | 0.0869 | 0.0254 | 0.1294 | 0.0202 | 0.0033 | 0.0368 | 0.0030 | 0.0000 | 0.0000 | 0.0077 | 0.1825 | 0.1176 | 0.0004 | 0.0021 | 0.0000 | 0.0000 | 0.0020 |
| 0.0021 | 0.0358 | 0.0836 | 0.0081 | 0.1086 | 0.0212 | 0.0180 | 0.0504 | 0.0016 | 0.0051 | 0.0000 | 0.0092 | 0.1063 | 0.0258 | 0.0000 | 0.0008 | 0.0000 | 0.0000 | 0.0024 |
| 0.0126 | 0.1192 | 0.1134 | 0.0549 | 0.0092 | 0.0282 | 0.0350 | 0.0539 | 0.0015 | 0.0049 | 0.0001 | 0.0227 | 0.0042 | 0.0132 | 0.0000 | 0.0015 | 0.0000 | 0.0000 | 0.0128 |
| 0.0000 | 0.0095 | 0.0236 | 0.0075 | 0.0646 | 0.0018 | 0.0045 | 0.0009 | 0.0001 | 0.0004 | 0.0000 | 0.0009 | 0.1102 | 0.0443 | 0.0005 | 0.0000 | 0.0000 | 0.0001 | 0.0021 |
| 0.0002 | 0.0153 | 0.0178 | 0.0065 | 0.0653 | 0.0108 | 0.0055 | 0.0253 | 0.0004 | 0.0000 | 0.0002 | 0.0039 | 0.0583 | 0.0079 | 0.0001 | 0.0002 | 0.0002 | 0.0002 | 0.0112 |
| 0.0017 | 0.0397 | 0.0097 | 0.0063 | 0.1012 | 0.0105 | 0.0015 | 0.0349 | 0.0007 | 0.0001 | 0.0014 | 0.0049 | 0.0423 | 0.0056 | 0.0001 | 0.0001 | 0.0006 | 0.0002 | 0.0158 |
| 0.0002 | 0.0242 | 0.0062 | 0.0328 | 0.0395 | 0.0078 | 0.0003 | 0.0381 | 0.0003 | 0.0005 | 0.0003 | 0.0086 | 0.0977 | 0.0220 | 0.0004 | 0.0005 | 0.0007 | 0.0001 | 0.0064 |
| 0.0016 | 0.0969 | 0.1415 | 0.0082 | 0.0516 | 0.0235 | 0.0222 | 0.0374 | 0.0030 | 0.0014 | 0.0001 | 0.0317 | 0.0798 | 0.0888 | 0.0004 | 0.0034 | 0.0001 | 0.0001 | 0.0041 |
| 0.0004 | 0.0590 | 0.0223 | 0.0163 | 0.1081 | 0.0426 | 0.0070 | 0.0805 | 0.0018 | 0.0502 | 0.0001 | 0.0048 | 0.1081 | 0.0124 | 0.0002 | 0.0001 | 0.0001 | 0.0002 | 0.0158 |
| 0.0000 | 0.0063 | 0.0085 | 0.0092 | 0.2060 | 0.0037 | 0.0023 | 0.0012 | 0.0000 | 0.0000 | 0.0000 | 0.0002 | 0.2060 | 0.0238 | 0.0002 | 0.0000 | 0.0000 | 0.0001 | 0.0064 |
| 0.0000 | 0.0079 | 0.0499 | 0.0055 | 0.2045 | 0.0060 | 0.0048 | 0.0036 | 0.0002 | 0.0013 | 0.0000 | 0.0004 | 0.2045 | 0.0288 | 0.0002 | 0.0000 | 0.0000 | 0.0001 | 0.0041 |
| 0.0000 | 0.0034 | 0.0065 | 0.0029 | 0.2061 | 0.0051 | 0.0009 | 0.0048 | 0.0000 | 0.0000 | 0.0001 | 0.0002 | 0.2061 | 0.0138 | 0.0000 | 0.0000 | 0.0001 | 0.0001 | 0.0023 |
| 0.0000 | 0.0043 | 0.0047 | 0.0032 | 0.2349 | 0.0060 | 0.0008 | 0.0089 | 0.0000 | 0.0000 | 0.0001 | 0.0003 | 0.2349 | 0.0112 | 0.0000 | 0.0000 | 0.0001 | 0.0002 | 0.0030 |
| 0.0011 | 0.0518 | 0.1505 | 0.0046 | 0.1712 | 0.0214 | 0.0022 | 0.0639 | 0.0050 | 0.0001 | 0.0002 | 0.0264 | 0.1712 | 0.0433 | 0.0001 | 0.0003 | 0.0002 | 0.0004 | 0.0038 |
| 0.0033 | 0.0653 | 0.0810 | 0.1172 | 0.0316 | 0.0160 | 0.0092 | 0.0136 | 0.0025 | 0.0150 | 0.0001 | 0.0225 | 0.0316 | 0.0543 | 0.0000 | 0.0014 | 0.0001 | 0.0001 | 0.0103 |
| 0.0000 | 0.0083 | 0.0434 | 0.0053 | 0.1858 | 0.0059 | 0.0042 | 0.0038 | 0.0001 | 0.0007 | 0.0002 | 0.0008 | 0.1858 | 0.0242 | 0.0001 | 0.0001 | 0.0002 | 0.0000 | 0.0034 |
| 0.0000 | 0.0058 | 0.0061 | 0.0418 | 0.1752 | 0.0061 | 0.0021 | 0.0043 | 0.0000 | 0.0002 | 0.0002 | 0.0010 | 0.1752 | 0.0073 | 0.0012 | 0.0000 | 0.0002 | 0.0001 | 0.0112 |
| 0.0032 | 0.0373 | 0.0661 | 0.0134 | 0.0680 | 0.0245 | 0.0027 | 0.0464 | 0.0026 | 0.0014 | 0.0000 | 0.0082 | 0.0680 | 0.0273 | 0.0001 | 0.0010 | 0.0000 | 0.0000 | 0.0069 |
| 0.0046 | 0.0240 | 0.0064 | 0.0329 | 0.2046 | 0.0412 | 0.0144 | 0.0645 | 0.0002 | 0.0027 | 0.0000 | 0.0091 | 0.2046 | 0.0089 | 0.0004 | 0.0001 | 0.0000 | 0.0001 | 0.0202 |
| 0.0095 | 0.0894 | 0.0996 | 0.0018 | 0.0915 | 0.0205 | 0.0488 | 0.0494 | 0.0030 | 0.0000 | 0.0001 | 0.0519 | 0.0915 | 0.0702 | 0.0000 | 0.0013 | 0.0001 | 0.0002 | 0.0018 |
| 0.0023 | 0.0794 | 0.0481 | 0.1162 | 0.0197 | 0.0125 | 0.0086 | 0.0100 | 0.0035 | 0.0065 | 0.0000 | 0.0240 | 0.0197 | 0.0544 | 0.0007 | 0.0014 | 0.0000 | 0.0000 | 0.0084 |
| 0.0000 | 0.0250 | 0.0091 | 0.0132 | 0.0250 | 0.0011 | 0.0012 | 0.0038 | 0.0000 | 0.0006 | 0.0000 | 0.0014 | 0.0250 | 0.0207 | 0.0000 | 0.0000 | 0.0000 | 0.0000 | 0.0049 |
| 0.0002 | 0.0152 | 0.0096 | 0.0122 | 0.0280 | 0.0032 | 0.0023 | 0.0110 | 0.0000 | 0.0003 | 0.0000 | 0.0008 | 0.0208 | 0.0056 | 0.0000 | 0.0001 | 0.0000 | 0.0003 | 0.0081 |
| 0.0000 | 0.0231 | 0.0178 | 0.0395 | 0.0672 | 0.0052 | 0.0053 | 0.0281 | 0.0000 | 0.0006 | 0.0001 | 0.0021 | 0.0731 | 0.0127 | 0.0000 | 0.0003 | 0.0001 | 0.0000 | 0.0222 |
| 0.0003 | 0.0206 | 0.0101 | 0.0300 | 0.0213 | 0.0045 | 0.0040 | 0.0169 | 0.0001 | 0.0002 | 0.0000 | 0.0018 | 0.0131 | 0.0049 | 0.0000 | 0.0002 | 0.0000 | 0.0002 | 0.0095 |
| 0.0000 | 0.0924 | 0.0182 | 0.0668 | 0.0186 | 0.0006 | 0.0075 | 0.0017 | 0.0003 | 0.0000 | 0.0000 | 0.0028 | 0.0262 | 0.0683 | 0.0000 | 0.0015 | 0.0000 | 0.0000 | 0.0101 |
| 0.0001 | 0.0206 | 0.0666 | 0.0096 | 0.0995 | 0.0021 | 0.0045 | 0.0047 | 0.0000 | 0.0001 | 0.0000 | 0.0023 | 0.2449 | 0.0667 | 0.0003 | 0.0009 | 0.0000 | 0.0000 | 0.0043 |
| 0.0001 | 0.0138 | 0.0332 | 0.0339 | 0.1172 | 0.0059 | 0.0045 | 0.0111 | 0.0005 | 0.0127 | 0.0000 | 0.0043 | 0.1245 | 0.0181 | 0.0000 | 0.0002 | 0.0000 | 0.0000 | 0.0022 |
| 0.0000 | 0.0185 | 0.0310 | 0.0492 | 0.1353 | 0.0034 | 0.0074 | 0.0026 | 0.0000 | 0.0000 | 0.0000 | 0.0008 | 0.3145 | 0.0451 | 0.0005 | 0.0005 | 0.0000 | 0.0000 | 0.0048 |
| 0.0000 | 0.0230 | 0.0173 | 0.0377 | 0.0648 | 0.0008 | 0.0025 | 0.0007 | 0.0000 | 0.0000 | 0.0000 | 0.0001 | 0.0398 | 0.0146 | 0.0000 | 0.0000 | 0.0000 | 0.0000 | 0.0043 |
| 0.0000 | 0.0125 | 0.0407 | 0.0285 | 0.1616 | 0.0023 | 0.0037 | 0.0013 | 0.0000 | 0.0000 | 0.0000 | 0.0004 | 0.1535 | 0.0215 | 0.0002 | 0.0001 | 0.0000 | 0.0000 | 0.0095 |
| 0.0000 | 0.0568 | 0.0282 | 0.0324 | 0.0124 | 0.0002 | 0.0012 | 0.0001 | 0.0000 | 0.0000 | 0.0000 | 0.0008 | 0.0157 | 0.0458 | 0.0002 | 0.0004 | 0.0000 | 0.0001 | 0.0064 |
| 0.0000 | 0.0699 | 0.0539 | 0.0180 | 0.0678 | 0.0019 | 0.0258 | 0.0017 | 0.0000 | 0.0000 | 0.0000 | 0.0002 | 0.0680 | 0.0429 | 0.0000 | 0.0001 | 0.0000 | 0.0000 | 0.0038 |
| 0.0001 | 0.0100 | 0.0116 | 0.0392 | 0.1203 | 0.0050 | 0.0031 | 0.0117 | 0.0004 | 0.0132 | 0.0000 | 0.0014 | 0.0599 | 0.0063 | 0.0000 | 0.0000 | 0.0000 | 0.0001 | 0.0056 |
| 0.0001 | 0.0154 | 0.0127 | 0.0232 | 0.0669 | 0.0038 | 0.0018 | 0.0082 | 0.0004 | 0.0025 | 0.0000 | 0.0027 | 0.0677 | 0.0140 | 0.0003 | 0.0003 | 0.0000 | 0.0000 | 0.0105 |
| 0.0001 | 0.0385 | 0.0079 | 0.0201 | 0.0557 | 0.0035 | 0.0015 | 0.0097 | 0.0011 | 0.0010 | 0.0000 | 0.0048 | 0.0458 | 0.0148 | 0.0001 | 0.0001 | 0.0000 | 0.0000 | 0.0084 |
| 0.0003 | 0.0987 | 0.0427 | 0.0309 | 0.0244 | 0.0032 | 0.0679 | 0.0051 | 0.0021 | 0.0012 | 0.0000 | 0.0615 | 0.0321 | 0.0683 | 0.0000 | 0.0012 | 0.0000 | 0.0000 | 0.0085 |
| 0.0001 | 0.0507 | 0.0530 | 0.0268 | 0.0537 | 0.0022 | 0.0374 | 0.0072 | 0.0006 | 0.0005 | 0.0001 | 0.0159 | 0.0627 | 0.0399 | 0.0000 | 0.0007 | 0.0001 | 0.0002 | 0.1570 |
| 0.0008 | 0.0061 | 0.0199 | 0.0116 | 0.0720 | 0.0126 | 0.0032 | 0.0158 | 0.0003 | 0.0092 | 0.0004 | 0.0014 | 0.0421 | 0.0047 | 0.0000 | 0.0002 | 0.0002 | 0.0003 | 0.0050 |
| 0.0014 | 0.0169 | 0.0328 | 0.0105 | 0.1233 | 0.0180 | 0.0088 | 0.0379 | 0.0019 | 0.0015 | 0.0024 | 0.0019 | 0.0544 | 0.0067 | 0.0003 | 0.0001 | 0.0010 | 0.0004 | 0.0038 |
| 0.0028 | 0.0155 | 0.0773 | 0.0060 | 0.0729 | 0.0125 | 0.0483 | 0.0217 | 0.0012 | 0.0003 | 0.0002 | 0.0069 | 0.0889 | 0.0124 | 0.0000 | 0.0009 | 0.0002 | 0.0000 | 0.0292 |
| 0.0004 | 0.0314 | 0.1281 | 0.0065 | 0.0920 | 0.0522 | 0.0129 | 0.0884 | 0.0068 | 0.0012 | 0.0001 | 0.0040 | 0.0727 | 0.0218 | 0.0000 | 0.0005 | 0.0001 | 0.0000 | 0.0169 |
| 0.0002 | 0.0220 | 0.0403 | 0.1752 | 0.1163 | 0.0608 | 0.0122 | 0.0451 | 0.0008 | 0.0101 | 0.0001 | 0.0016 | 0.1028 | 0.0176 | 0.0001 | 0.0001 | 0.0001 | 0.0000 | 0.0148 |
| 0.0000 | 0.0087 | 0.0423 | 0.0177 | 0.1228 | 0.0042 | 0.0067 | 0.0023 | 0.0003 | 0.0018 | 0.0001 | 0.0017 | 0.4352 | 0.1010 | 0.0003 | 0.0012 | 0.0003 | 0.0001 | 0.0014 |
| 0.0010 | 0.0387 | 0.0912 | 0.0065 | 0.0843 | 0.0144 | 0.0239 | 0.0208 | 0.0027 | 0.0007 | 0.0003 | 0.0032 | 0.0512 | 0.0232 | 0.0000 | 0.0010 | 0.0002 | 0.0003 | 0.0117 |
| 0.0001 | 0.0082 | 0.0120 | 0.0041 | 0.2431 | 0.0154 | 0.0014 | 0.0326 | 0.0004 | 0.0014 | 0.0003 | 0.0005 | 0.0857 | 0.0031 | 0.0001 | 0.0000 | 0.0001 | 0.0003 | 0.0077 |
| 0.0009 | 0.0538 | 0.0366 | 0.0104 | 0.0541 | 0.0153 | 0.0074 | 0.0263 | 0.0018 | 0.0027 | 0.0000 | 0.0245 | 0.0791 | 0.0492 | 0.0004 | 0.0010 | 0.0001 | 0.0000 | 0.0030 |
| 0.0012 | 0.0210 | 0.0614 | 0.0044 | 0.1530 | 0.0042 | 0.0053 | 0.0046 | 0.0003 | 0.0039 | 0.0000 | 0.0035 | 0.1016 | 0.0205 | 0.0002 | 0.0004 | 0.0000 | 0.0000 | 0.0221 |
| 0.0000 | 0.0148 | 0.0239 | 0.0111 | 0.0555 | 0.0183 | 0.0051 | 0.0236 | 0.0010 | 0.0040 | 0.0003 | 0.0035 | 0.0629 | 0.0120 | 0.0002 | 0.0005 | 0.0004 | 0.0000 | 0.0026 |
| 0.0019 | 0.1053 | 0.0445 | 0.0035 | 0.0552 | 0.0072 | 0.0098 | 0.0265 | 0.0024 | 0.0000 | 0.0002 | 0.0245 | 0.0505 | 0.0643 | 0.0000 | 0.0018 | 0.0002 | 0.0000 | 0.0062 |
| 0.0001 | 0.0299 | 0.0065 | 0.0135 | 0.0104 | 0.0052 | 0.0015 | 0.0199 | 0.0014 | 0.0002 | 0.0001 | 0.0083 | 0.0190 | 0.0243 | 0.0000 | 0.0003 | 0.0001 | 0.0000 | 0.0015 |
| 0.0010 | 0.0437 | 0.0470 | 0.0053 | 0.0086 | 0.0035 | 0.0095 | 0.0113 | 0.0009 | 0.0006 | 0.0000 | 0.0222 | 0.0085 | 0.0177 | 0.0000 | 0.0009 | 0.0000 | 0.0000 | 0.0090 |
| 0.0001 | 0.0175 | 0.0078 | 0.0337 | 0.0204 | 0.0005 | 0.0022 | 0.0028 | 0.0001 | 0.0005 | 0.0001 | 0.0021 | 0.0213 | 0.0141 | 0.0000 | 0.0001 | 0.0001 | 0.0001 | 0.0104 |
| 0.0008 | 0.0276 | 0.0732 | 0.0321 | 0.0056 | 0.0083 | 0.0116 | 0.0211 | 0.0006 | 0.0017 | 0.0000 | 0.0089 | 0.0040 | 0.0138 | 0.0000 | 0.0007 | 0.0000 | 0.0000 | 0.0044 |

**Supplementary Table 3: Key Bacterial Feature Relative Abundance for Male Mice.** Heading identifies lowest taxonomic level. Table includes all designated phyla and features with a significant model.
