# Supplementary material for: Microbiome variations induced by delta9-tetrahydrocannabinol predict weight reduction in obese mice
Source: Front Microbiomes. 2024 Jul 16;3:1412468. doi: 10.3389/frmbi.2024.1412468 (PMC12993608; doi:10.3389/frmbi.2024.1412468)
Supplement: Supplementary file 10 [file Table_4.docx]

| ratid | day | final_treatment | *p__Proteobacteria* | *p__Firmicutes* | *p__Actinobacteria* | *p__Bacteroidetes* | *p__Deferribacteres* | *p__TM7* | *p__Tenericutes* | *c__Alphaproteobacteria* | *c__Clostridia* | *c__Actinobacteria* | *c__Coriobacteriia* | *c__TM7-3* | *c__Mollicutes* |
| --- | --- | --- | --- | --- | --- | --- | --- | --- | --- | --- | --- | --- | --- | --- | --- |
| 7 | 1 | THC | 0.1695 | 0.5277 | 0.0099 | 0.1610 | 0.1266 | 0.0000 | 0.0044 | 0.0052 | 0.2521 | 0.0006 | 0.0093 | 0.0000 | 0.0044 |
| 7 | 2 | THC | 0.0871 | 0.5718 | 0.0266 | 0.3078 | 0.0064 | 0.0000 | 0.0003 | 0.0003 | 0.1591 | 0.0001 | 0.0265 | 0.0000 | 0.0003 |
| 7 | 3 | THC | 0.0566 | 0.7099 | 0.0327 | 0.1977 | 0.0028 | 0.0000 | 0.0002 | 0.0001 | 0.0780 | 0.0001 | 0.0327 | 0.0000 | 0.0002 |
| 7 | 4 | THC | 0.0095 | 0.8640 | 0.0200 | 0.1036 | 0.0009 | 0.0000 | 0.0020 | 0.0002 | 0.1796 | 0.0007 | 0.0192 | 0.0000 | 0.0020 |
| 8 | 1 | VEH | 0.0231 | 0.7322 | 0.0272 | 0.2064 | 0.0027 | 0.0008 | 0.0076 | 0.0003 | 0.2168 | 0.0002 | 0.0270 | 0.0008 | 0.0076 |
| 8 | 2 | VEH | 0.0302 | 0.5926 | 0.0361 | 0.3385 | 0.0007 | 0.0000 | 0.0018 | 0.0000 | 0.1283 | 0.0001 | 0.0360 | 0.0000 | 0.0018 |
| 8 | 3 | VEH | 0.0201 | 0.6655 | 0.0325 | 0.2729 | 0.0021 | 0.0010 | 0.0057 | 0.0002 | 0.2846 | 0.0001 | 0.0324 | 0.0010 | 0.0057 |
| 8 | 4 | VEH | 0.1440 | 0.5936 | 0.0316 | 0.1124 | 0.1108 | 0.0004 | 0.0057 | 0.0219 | 0.2418 | 0.0003 | 0.0313 | 0.0004 | 0.0057 |
| 8 | 9 | VEH | 0.1416 | 0.7096 | 0.0269 | 0.0958 | 0.0234 | 0.0002 | 0.0014 | 0.0081 | 0.4782 | 0.0001 | 0.0268 | 0.0002 | 0.0014 |
| 8 | 15 | VEH | 0.0168 | 0.7464 | 0.0903 | 0.1444 | 0.0015 | 0.0000 | 0.0006 | 0.0000 | 0.1068 | 0.0000 | 0.0903 | 0.0000 | 0.0006 |
| 9 | 1 | VEH | 0.0453 | 0.7642 | 0.0449 | 0.1440 | 0.0009 | 0.0000 | 0.0005 | 0.0003 | 0.1416 | 0.0003 | 0.0446 | 0.0000 | 0.0005 |
| 9 | 2 | VEH | 0.0599 | 0.6207 | 0.0164 | 0.2984 | 0.0026 | 0.0002 | 0.0013 | 0.0014 | 0.3208 | 0.0003 | 0.0161 | 0.0002 | 0.0013 |
| 9 | 3 | VEH | 0.0151 | 0.7099 | 0.0696 | 0.2016 | 0.0007 | 0.0002 | 0.0025 | 0.0000 | 0.1408 | 0.0003 | 0.0692 | 0.0002 | 0.0025 |
| 9 | 4 | VEH | 0.1639 | 0.6254 | 0.0313 | 0.1541 | 0.0226 | 0.0002 | 0.0012 | 0.0056 | 0.3388 | 0.0002 | 0.0311 | 0.0002 | 0.0012 |
| 9 | 9 | VEH | 0.0178 | 0.8129 | 0.0708 | 0.0938 | 0.0024 | 0.0002 | 0.0020 | 0.0001 | 0.1594 | 0.0004 | 0.0704 | 0.0002 | 0.0020 |
| 9 | 15 | VEH | 0.0509 | 0.6491 | 0.0693 | 0.2276 | 0.0023 | 0.0000 | 0.0006 | 0.0000 | 0.1210 | 0.0003 | 0.0690 | 0.0000 | 0.0006 |
| 10 | 1 | THC | 0.0426 | 0.7703 | 0.0372 | 0.1434 | 0.0055 | 0.0002 | 0.0006 | 0.0011 | 0.1515 | 0.0003 | 0.0369 | 0.0002 | 0.0006 |
| 10 | 2 | THC | 0.0364 | 0.5686 | 0.0379 | 0.3448 | 0.0100 | 0.0002 | 0.0017 | 0.0008 | 0.1453 | 0.0003 | 0.0376 | 0.0002 | 0.0017 |
| 10 | 3 | THC | 0.0166 | 0.7004 | 0.0239 | 0.2573 | 0.0005 | 0.0000 | 0.0004 | 0.0001 | 0.0623 | 0.0004 | 0.0234 | 0.0000 | 0.0004 |
| 10 | 4 | THC | 0.0406 | 0.8344 | 0.0102 | 0.1068 | 0.0025 | 0.0000 | 0.0054 | 0.0005 | 0.0953 | 0.0004 | 0.0098 | 0.0000 | 0.0054 |
| 10 | 9 | THC | 0.0453 | 0.8544 | 0.0066 | 0.0907 | 0.0029 | 0.0000 | 0.0000 | 0.0000 | 0.1623 | 0.0002 | 0.0064 | 0.0000 | 0.0000 |
| 10 | 15 | THC | 0.2219 | 0.5536 | 0.0105 | 0.1500 | 0.0578 | 0.0002 | 0.0046 | 0.0042 | 0.2677 | 0.0002 | 0.0103 | 0.0002 | 0.0046 |
| 11 | 1 | THC | 0.0200 | 0.6977 | 0.0045 | 0.2657 | 0.0021 | 0.0004 | 0.0096 | 0.0000 | 0.1822 | 0.0003 | 0.0041 | 0.0004 | 0.0096 |
| 11 | 2 | THC | 0.0348 | 0.7103 | 0.0062 | 0.2459 | 0.0014 | 0.0000 | 0.0011 | 0.0000 | 0.0848 | 0.0002 | 0.0060 | 0.0000 | 0.0011 |
| 11 | 3 | THC | 0.0275 | 0.6768 | 0.0069 | 0.2849 | 0.0018 | 0.0000 | 0.0012 | 0.0000 | 0.0608 | 0.0003 | 0.0066 | 0.0000 | 0.0012 |
| 11 | 4 | THC | 0.0296 | 0.7563 | 0.0078 | 0.2034 | 0.0010 | 0.0000 | 0.0018 | 0.0000 | 0.1139 | 0.0003 | 0.0075 | 0.0000 | 0.0018 |
| 11 | 9 | THC | 0.0280 | 0.8347 | 0.0280 | 0.1067 | 0.0008 | 0.0000 | 0.0018 | 0.0002 | 0.0781 | 0.0005 | 0.0275 | 0.0000 | 0.0018 |
| 11 | 15 | THC | 0.0141 | 0.8033 | 0.0024 | 0.1707 | 0.0032 | 0.0009 | 0.0054 | 0.0005 | 0.3313 | 0.0003 | 0.0022 | 0.0009 | 0.0054 |
| 12 | 1 | VEH | 0.0164 | 0.9223 | 0.0053 | 0.0551 | 0.0008 | 0.0000 | 0.0000 | 0.0000 | 0.3194 | 0.0000 | 0.0053 | 0.0000 | 0.0000 |
| 12 | 3 | VEH | 0.0199 | 0.6456 | 0.0300 | 0.2946 | 0.0019 | 0.0008 | 0.0070 | 0.0002 | 0.2892 | 0.0003 | 0.0297 | 0.0008 | 0.0070 |
| 12 | 4 | VEH | 0.0423 | 0.6873 | 0.0336 | 0.2224 | 0.0123 | 0.0002 | 0.0017 | 0.0017 | 0.1196 | 0.0002 | 0.0334 | 0.0002 | 0.0017 |
| 12 | 9 | VEH | 0.1551 | 0.6401 | 0.0436 | 0.1485 | 0.0104 | 0.0000 | 0.0015 | 0.0020 | 0.2629 | 0.0002 | 0.0435 | 0.0000 | 0.0015 |
| 19 | 1 | THC | 0.1192 | 0.6511 | 0.0053 | 0.1756 | 0.0456 | 0.0001 | 0.0004 | 0.0027 | 0.3492 | 0.0000 | 0.0053 | 0.0001 | 0.0004 |
| 19 | 2 | THC | 0.0348 | 0.7723 | 0.0147 | 0.1759 | 0.0010 | 0.0000 | 0.0012 | 0.0005 | 0.2924 | 0.0000 | 0.0147 | 0.0000 | 0.0012 |
| 19 | 3 | THC | 0.0979 | 0.7161 | 0.0101 | 0.1611 | 0.0118 | 0.0000 | 0.0024 | 0.0005 | 0.2003 | 0.0000 | 0.0101 | 0.0000 | 0.0024 |
| 19 | 4 | THC | 0.0500 | 0.7447 | 0.0166 | 0.1829 | 0.0016 | 0.0001 | 0.0041 | 0.0003 | 0.1562 | 0.0000 | 0.0166 | 0.0001 | 0.0041 |
| 19 | 9 | THC | 0.0339 | 0.5830 | 0.0539 | 0.2988 | 0.0292 | 0.0001 | 0.0007 | 0.0005 | 0.1431 | 0.0000 | 0.0539 | 0.0001 | 0.0007 |
| 19 | 15 | THC | 0.2601 | 0.2192 | 0.1481 | 0.2353 | 0.0036 | 0.0048 | 0.0053 | 0.1578 | 0.0974 | 0.1132 | 0.0171 | 0.0004 | 0.0053 |
| 20 | 1 | THC | 0.0637 | 0.6922 | 0.0636 | 0.1730 | 0.0063 | 0.0000 | 0.0010 | 0.0000 | 0.1756 | 0.0003 | 0.0632 | 0.0000 | 0.0010 |
| 20 | 2 | THC | 0.1191 | 0.7030 | 0.0149 | 0.1432 | 0.0193 | 0.0000 | 0.0002 | 0.0009 | 0.2939 | 0.0000 | 0.0149 | 0.0000 | 0.0002 |
| 20 | 3 | THC | 0.1757 | 0.5946 | 0.0052 | 0.2105 | 0.0135 | 0.0000 | 0.0004 | 0.0076 | 0.3454 | 0.0000 | 0.0052 | 0.0000 | 0.0004 |
| 20 | 4 | THC | 0.0336 | 0.6073 | 0.0103 | 0.3355 | 0.0128 | 0.0000 | 0.0003 | 0.0023 | 0.1940 | 0.0000 | 0.0103 | 0.0000 | 0.0003 |
| 20 | 9 | THC | 0.0418 | 0.4553 | 0.0038 | 0.4391 | 0.0439 | 0.0000 | 0.0153 | 0.0013 | 0.3134 | 0.0000 | 0.0038 | 0.0000 | 0.0153 |
| 20 | 15 | THC | 0.2273 | 0.5649 | 0.0010 | 0.1643 | 0.0399 | 0.0000 | 0.0014 | 0.0009 | 0.3056 | 0.0000 | 0.0010 | 0.0000 | 0.0014 |
| 21 | 1 | THC | 0.0866 | 0.7586 | 0.0098 | 0.1193 | 0.0217 | 0.0000 | 0.0036 | 0.0002 | 0.1797 | 0.0000 | 0.0098 | 0.0000 | 0.0036 |
| 21 | 2 | THC | 0.1124 | 0.5919 | 0.0093 | 0.2713 | 0.0134 | 0.0000 | 0.0015 | 0.0152 | 0.2269 | 0.0000 | 0.0093 | 0.0000 | 0.0015 |
| 21 | 3 | THC | 0.0763 | 0.6909 | 0.0082 | 0.2178 | 0.0064 | 0.0000 | 0.0003 | 0.0028 | 0.2458 | 0.0000 | 0.0082 | 0.0000 | 0.0003 |
| 21 | 4 | THC | 0.0308 | 0.6809 | 0.0120 | 0.2535 | 0.0213 | 0.0000 | 0.0007 | 0.0016 | 0.2292 | 0.0000 | 0.0120 | 0.0000 | 0.0007 |
| 21 | 9 | THC | 0.0393 | 0.5827 | 0.0176 | 0.3497 | 0.0104 | 0.0001 | 0.0000 | 0.0006 | 0.2152 | 0.0000 | 0.0176 | 0.0001 | 0.0000 |
| 21 | 15 | THC | 0.0496 | 0.6225 | 0.0075 | 0.3181 | 0.0016 | 0.0000 | 0.0007 | 0.0006 | 0.1096 | 0.0001 | 0.0074 | 0.0000 | 0.0007 |
| 22 | 1 | VEH | 0.0507 | 0.6042 | 0.0518 | 0.2861 | 0.0025 | 0.0003 | 0.0043 | 0.0006 | 0.2947 | 0.0000 | 0.0518 | 0.0003 | 0.0043 |
| 22 | 2 | VEH | 0.1810 | 0.6493 | 0.0025 | 0.1114 | 0.0551 | 0.0000 | 0.0005 | 0.0001 | 0.4914 | 0.0000 | 0.0025 | 0.0000 | 0.0005 |
| 22 | 3 | VEH | 0.2420 | 0.5343 | 0.0041 | 0.1254 | 0.0898 | 0.0000 | 0.0039 | 0.0000 | 0.3049 | 0.0000 | 0.0041 | 0.0000 | 0.0039 |
| 22 | 4 | VEH | 0.0298 | 0.6249 | 0.0990 | 0.2430 | 0.0018 | 0.0001 | 0.0013 | 0.0006 | 0.1457 | 0.0001 | 0.0989 | 0.0001 | 0.0013 |
| 22 | 9 | VEH | 0.1907 | 0.5506 | 0.0331 | 0.1968 | 0.0263 | 0.0001 | 0.0017 | 0.0037 | 0.3896 | 0.0000 | 0.0331 | 0.0001 | 0.0017 |
| 22 | 15 | VEH | 0.0705 | 0.5082 | 0.0471 | 0.3593 | 0.0053 | 0.0001 | 0.0088 | 0.0029 | 0.2420 | 0.0000 | 0.0471 | 0.0001 | 0.0088 |
| 23 | 1 | VEH | 0.0283 | 0.7164 | 0.0170 | 0.2313 | 0.0016 | 0.0001 | 0.0053 | 0.0005 | 0.1604 | 0.0000 | 0.0170 | 0.0001 | 0.0053 |
| 23 | 2 | VEH | 0.0716 | 0.7039 | 0.0165 | 0.1960 | 0.0100 | 0.0001 | 0.0014 | 0.0018 | 0.2080 | 0.0000 | 0.0165 | 0.0001 | 0.0014 |
| 23 | 3 | VEH | 0.0521 | 0.5482 | 0.0368 | 0.3514 | 0.0064 | 0.0003 | 0.0048 | 0.0003 | 0.2831 | 0.0000 | 0.0368 | 0.0003 | 0.0048 |
| 23 | 4 | VEH | 0.2079 | 0.5611 | 0.0057 | 0.2012 | 0.0214 | 0.0001 | 0.0007 | 0.0038 | 0.3632 | 0.0000 | 0.0056 | 0.0001 | 0.0007 |
| 23 | 9 | VEH | 0.1268 | 0.5382 | 0.0438 | 0.2511 | 0.0386 | 0.0001 | 0.0006 | 0.0053 | 0.2830 | 0.0000 | 0.0438 | 0.0001 | 0.0006 |
| 23 | 15 | VEH | 0.0750 | 0.4803 | 0.0430 | 0.3826 | 0.0081 | 0.0001 | 0.0103 | 0.0034 | 0.2376 | 0.0000 | 0.0430 | 0.0001 | 0.0103 |
| 24 | 1 | VEH | 0.1854 | 0.6019 | 0.0128 | 0.1874 | 0.0098 | 0.0003 | 0.0017 | 0.0042 | 0.4878 | 0.0000 | 0.0128 | 0.0003 | 0.0017 |
| 24 | 2 | VEH | 0.0655 | 0.6540 | 0.0730 | 0.1984 | 0.0059 | 0.0007 | 0.0024 | 0.0007 | 0.2664 | 0.0000 | 0.0730 | 0.0007 | 0.0024 |
| 24 | 3 | VEH | 0.0371 | 0.7161 | 0.0663 | 0.1760 | 0.0013 | 0.0003 | 0.0029 | 0.0001 | 0.2765 | 0.0000 | 0.0663 | 0.0003 | 0.0029 |
| 24 | 9 | VEH | 0.0565 | 0.6448 | 0.0694 | 0.2240 | 0.0032 | 0.0003 | 0.0013 | 0.0075 | 0.2796 | 0.0000 | 0.0694 | 0.0003 | 0.0013 |
| 24 | 15 | VEH | 0.2216 | 0.5521 | 0.0076 | 0.1834 | 0.0312 | 0.0002 | 0.0028 | 0.0211 | 0.3988 | 0.0000 | 0.0076 | 0.0002 | 0.0028 |

| *o__Clostridiales* | *o__Actinomycetales* | *o__Coriobacteriales* | *o__CW040* | *o__Rickettsiales* | *o__RF39* | *f__Ruminococcaceae* | *f__Lactobacillaceae* | *f__Rikenellaceae* | *f__Bacteroidaceae* | *f__F16* | *g__Ruminococcus* | *g__Lactobacillus* | *g__Ruminococcus* | *g__Coprococcus* | *g__Lactobacillus;s__salivarius* |
| --- | --- | --- | --- | --- | --- | --- | --- | --- | --- | --- | --- | --- | --- | --- | --- |
| 0.2521 | 0.0006 | 0.0093 | 0.0000 | 0.0052 | 0.0002 | 0.0405 | 0.0310 | 0.0088 | 0.0909 | 0.0000 | 0.0360 | 0.0310 | 0.0105 | 0.0050 | 0.0039 |
| 0.1591 | 0.0001 | 0.0265 | 0.0000 | 0.0003 | 0.0001 | 0.0162 | 0.0667 | 0.0076 | 0.2080 | 0.0000 | 0.0396 | 0.0667 | 0.0019 | 0.0036 | 0.0090 |
| 0.0780 | 0.0001 | 0.0327 | 0.0000 | 0.0001 | 0.0002 | 0.0051 | 0.0548 | 0.0043 | 0.1349 | 0.0000 | 0.0160 | 0.0548 | 0.0002 | 0.0012 | 0.0297 |
| 0.1796 | 0.0007 | 0.0192 | 0.0000 | 0.0002 | 0.0000 | 0.0151 | 0.0270 | 0.0031 | 0.0549 | 0.0000 | 0.0112 | 0.0270 | 0.0022 | 0.0016 | 0.0084 |
| 0.2168 | 0.0002 | 0.0270 | 0.0008 | 0.0004 | 0.0014 | 0.0480 | 0.1561 | 0.0367 | 0.0729 | 0.0008 | 0.0269 | 0.1561 | 0.0078 | 0.0039 | 0.0123 |
| 0.1283 | 0.0001 | 0.0360 | 0.0000 | 0.0000 | 0.0007 | 0.0434 | 0.0272 | 0.0099 | 0.1766 | 0.0000 | 0.0123 | 0.0272 | 0.0013 | 0.0009 | 0.0050 |
| 0.2846 | 0.0001 | 0.0324 | 0.0010 | 0.0002 | 0.0036 | 0.0738 | 0.0365 | 0.0180 | 0.1471 | 0.0010 | 0.0344 | 0.0365 | 0.0066 | 0.0036 | 0.0171 |
| 0.2418 | 0.0003 | 0.0313 | 0.0004 | 0.0219 | 0.0010 | 0.0808 | 0.0395 | 0.0217 | 0.0318 | 0.0004 | 0.0254 | 0.0395 | 0.0527 | 0.0030 | 0.0080 |
| 0.4782 | 0.0001 | 0.0268 | 0.0002 | 0.0081 | 0.0009 | 0.1642 | 0.0431 | 0.0181 | 0.0335 | 0.0002 | 0.0665 | 0.0431 | 0.0338 | 0.0101 | 0.0076 |
| 0.1068 | 0.0000 | 0.0903 | 0.0000 | 0.0000 | 0.0001 | 0.0288 | 0.1298 | 0.0117 | 0.0829 | 0.0000 | 0.0188 | 0.1298 | 0.0023 | 0.0018 | 0.0095 |
| 0.1416 | 0.0003 | 0.0446 | 0.0000 | 0.0003 | 0.0005 | 0.0396 | 0.1035 | 0.0097 | 0.0633 | 0.0000 | 0.0201 | 0.1035 | 0.0031 | 0.0023 | 0.0256 |
| 0.3208 | 0.0003 | 0.0161 | 0.0002 | 0.0014 | 0.0006 | 0.0680 | 0.0241 | 0.0226 | 0.1604 | 0.0002 | 0.0643 | 0.0241 | 0.0055 | 0.0070 | 0.0074 |
| 0.1408 | 0.0003 | 0.0692 | 0.0002 | 0.0000 | 0.0025 | 0.0267 | 0.0268 | 0.0077 | 0.1135 | 0.0002 | 0.0143 | 0.0268 | 0.0029 | 0.0010 | 0.0165 |
| 0.3388 | 0.0002 | 0.0311 | 0.0002 | 0.0056 | 0.0011 | 0.0944 | 0.0344 | 0.0163 | 0.0683 | 0.0002 | 0.0598 | 0.0343 | 0.0185 | 0.0059 | 0.0109 |
| 0.1594 | 0.0004 | 0.0704 | 0.0002 | 0.0001 | 0.0019 | 0.0390 | 0.1751 | 0.0085 | 0.0283 | 0.0002 | 0.0151 | 0.1751 | 0.0026 | 0.0013 | 0.0308 |
| 0.1210 | 0.0003 | 0.0690 | 0.0000 | 0.0000 | 0.0001 | 0.0538 | 0.1040 | 0.0358 | 0.0954 | 0.0000 | 0.0075 | 0.1040 | 0.0022 | 0.0006 | 0.0412 |
| 0.1515 | 0.0003 | 0.0369 | 0.0002 | 0.0001 | 0.0006 | 0.0154 | 0.0233 | 0.0135 | 0.0557 | 0.0002 | 0.0109 | 0.0233 | 0.0019 | 0.0027 | 0.0066 |
| 0.1453 | 0.0003 | 0.0376 | 0.0002 | 0.0008 | 0.0017 | 0.0229 | 0.0150 | 0.0266 | 0.1790 | 0.0002 | 0.0086 | 0.0150 | 0.0041 | 0.0007 | 0.0087 |
| 0.0623 | 0.0003 | 0.0234 | 0.0000 | 0.0000 | 0.0001 | 0.0038 | 0.0654 | 0.0072 | 0.1741 | 0.0000 | 0.0044 | 0.0653 | 0.0006 | 0.0003 | 0.0069 |
| 0.0953 | 0.0002 | 0.0098 | 0.0000 | 0.0005 | 0.0006 | 0.0064 | 0.1199 | 0.0076 | 0.0635 | 0.0000 | 0.0121 | 0.1198 | 0.0016 | 0.0019 | 0.0051 |
| 0.1623 | 0.0002 | 0.0064 | 0.0000 | 0.0000 | 0.0000 | 0.0404 | 0.0325 | 0.0015 | 0.0658 | 0.0000 | 0.0258 | 0.0325 | 0.0005 | 0.0048 | 0.0066 |
| 0.2677 | 0.0002 | 0.0103 | 0.0002 | 0.0037 | 0.0025 | 0.0464 | 0.0137 | 0.0199 | 0.0603 | 0.0002 | 0.0252 | 0.0137 | 0.0089 | 0.0061 | 0.0030 |
| 0.1822 | 0.0003 | 0.0041 | 0.0004 | 0.0000 | 0.0010 | 0.0191 | 0.0656 | 0.0177 | 0.1146 | 0.0004 | 0.0317 | 0.0656 | 0.0014 | 0.0122 | 0.0104 |
| 0.0848 | 0.0002 | 0.0060 | 0.0000 | 0.0000 | 0.0001 | 0.0122 | 0.0720 | 0.0098 | 0.1441 | 0.0000 | 0.0216 | 0.0719 | 0.0007 | 0.0148 | 0.0210 |
| 0.0608 | 0.0003 | 0.0066 | 0.0000 | 0.0000 | 0.0001 | 0.0113 | 0.0864 | 0.0095 | 0.1684 | 0.0000 | 0.0136 | 0.0864 | 0.0005 | 0.0099 | 0.0248 |
| 0.1139 | 0.0003 | 0.0075 | 0.0000 | 0.0000 | 0.0017 | 0.0104 | 0.0154 | 0.0043 | 0.1307 | 0.0000 | 0.0206 | 0.0154 | 0.0009 | 0.0142 | 0.0033 |
| 0.0781 | 0.0005 | 0.0275 | 0.0000 | 0.0000 | 0.0013 | 0.0085 | 0.0649 | 0.0031 | 0.0775 | 0.0000 | 0.0109 | 0.0648 | 0.0004 | 0.0131 | 0.0102 |
| 0.3313 | 0.0003 | 0.0022 | 0.0009 | 0.0005 | 0.0054 | 0.0651 | 0.1193 | 0.0175 | 0.0420 | 0.0009 | 0.0487 | 0.1193 | 0.0063 | 0.0709 | 0.0177 |
| 0.3194 | 0.0000 | 0.0053 | 0.0000 | 0.0000 | 0.0000 | 0.0922 | 0.0936 | 0.0016 | 0.0339 | 0.0000 | 0.0194 | 0.0936 | 0.0004 | 0.0030 | 0.0092 |
| 0.2892 | 0.0003 | 0.0297 | 0.0008 | 0.0002 | 0.0043 | 0.0692 | 0.0386 | 0.0182 | 0.1605 | 0.0008 | 0.0352 | 0.0385 | 0.0065 | 0.0040 | 0.0163 |
| 0.1196 | 0.0002 | 0.0334 | 0.0002 | 0.0017 | 0.0001 | 0.0179 | 0.0374 | 0.0142 | 0.0920 | 0.0002 | 0.0131 | 0.0374 | 0.0033 | 0.0018 | 0.0046 |
| 0.2629 | 0.0002 | 0.0435 | 0.0000 | 0.0020 | 0.0007 | 0.0503 | 0.0406 | 0.0173 | 0.0458 | 0.0000 | 0.0229 | 0.0406 | 0.0089 | 0.0039 | 0.0046 |
| 0.3492 | 0.0000 | 0.0053 | 0.0001 | 0.0007 | 0.0000 | 0.0853 | 0.0380 | 0.0427 | 0.0141 | 0.0001 | 0.0262 | 0.0380 | 0.0189 | 0.0045 | 0.0216 |
| 0.2924 | 0.0000 | 0.0147 | 0.0000 | 0.0000 | 0.0000 | 0.0677 | 0.1190 | 0.0136 | 0.0052 | 0.0000 | 0.0132 | 0.1189 | 0.0054 | 0.0034 | 0.0228 |
| 0.2003 | 0.0000 | 0.0101 | 0.0000 | 0.0004 | 0.0000 | 0.0348 | 0.0448 | 0.0145 | 0.0109 | 0.0000 | 0.0152 | 0.0448 | 0.0086 | 0.0040 | 0.0113 |
| 0.1562 | 0.0000 | 0.0166 | 0.0001 | 0.0002 | 0.0000 | 0.0237 | 0.0592 | 0.0267 | 0.0280 | 0.0001 | 0.0050 | 0.0592 | 0.0033 | 0.0014 | 0.0090 |
| 0.1431 | 0.0000 | 0.0539 | 0.0001 | 0.0000 | 0.0000 | 0.0424 | 0.0154 | 0.0232 | 0.0442 | 0.0001 | 0.0060 | 0.0154 | 0.0078 | 0.0008 | 0.0134 |
| 0.0973 | 0.1130 | 0.0171 | 0.0000 | 0.0014 | 0.0001 | 0.0222 | 0.0297 | 0.0331 | 0.0194 | 0.0000 | 0.0070 | 0.0297 | 0.0025 | 0.0009 | 0.0059 |
| 0.1756 | 0.0000 | 0.0632 | 0.0000 | 0.0000 | 0.0000 | 0.0124 | 0.0135 | 0.0044 | 0.1061 | 0.0000 | 0.0319 | 0.0135 | 0.0012 | 0.0087 | 0.0040 |
| 0.2939 | 0.0000 | 0.0149 | 0.0000 | 0.0003 | 0.0000 | 0.0642 | 0.0060 | 0.0118 | 0.0266 | 0.0000 | 0.0414 | 0.0059 | 0.0115 | 0.0057 | 0.0041 |
| 0.3454 | 0.0000 | 0.0052 | 0.0000 | 0.0000 | 0.0000 | 0.0849 | 0.0081 | 0.0295 | 0.0091 | 0.0000 | 0.0432 | 0.0081 | 0.0077 | 0.0086 | 0.0042 |
| 0.1940 | 0.0000 | 0.0103 | 0.0000 | 0.0001 | 0.0000 | 0.0761 | 0.0090 | 0.0306 | 0.0111 | 0.0000 | 0.0121 | 0.0090 | 0.0060 | 0.0012 | 0.0083 |
| 0.3134 | 0.0000 | 0.0038 | 0.0000 | 0.0003 | 0.0000 | 0.1213 | 0.0079 | 0.0505 | 0.0620 | 0.0000 | 0.0130 | 0.0079 | 0.0533 | 0.0018 | 0.0045 |
| 0.3056 | 0.0000 | 0.0010 | 0.0000 | 0.0002 | 0.0000 | 0.0848 | 0.0084 | 0.0365 | 0.0156 | 0.0000 | 0.0359 | 0.0084 | 0.0303 | 0.0043 | 0.0043 |
| 0.1797 | 0.0000 | 0.0098 | 0.0000 | 0.0000 | 0.0000 | 0.0331 | 0.0149 | 0.0432 | 0.0033 | 0.0000 | 0.0305 | 0.0149 | 0.0058 | 0.0018 | 0.0109 |
| 0.2269 | 0.0000 | 0.0093 | 0.0000 | 0.0001 | 0.0000 | 0.0461 | 0.0067 | 0.0168 | 0.0632 | 0.0000 | 0.0244 | 0.0067 | 0.0054 | 0.0031 | 0.0054 |
| 0.2458 | 0.0000 | 0.0082 | 0.0000 | 0.0002 | 0.0000 | 0.0468 | 0.0085 | 0.0101 | 0.0357 | 0.0000 | 0.0313 | 0.0085 | 0.0061 | 0.0056 | 0.0076 |
| 0.2292 | 0.0000 | 0.0120 | 0.0000 | 0.0005 | 0.0000 | 0.0423 | 0.0085 | 0.0367 | 0.0101 | 0.0000 | 0.0151 | 0.0085 | 0.0072 | 0.0022 | 0.0068 |
| 0.2152 | 0.0000 | 0.0176 | 0.0001 | 0.0003 | 0.0000 | 0.0600 | 0.0109 | 0.0390 | 0.0279 | 0.0001 | 0.0085 | 0.0109 | 0.0211 | 0.0010 | 0.0096 |
| 0.1096 | 0.0001 | 0.0074 | 0.0000 | 0.0000 | 0.0000 | 0.0453 | 0.0072 | 0.0121 | 0.0548 | 0.0000 | 0.0071 | 0.0072 | 0.0032 | 0.0014 | 0.0048 |
| 0.2947 | 0.0000 | 0.0518 | 0.0003 | 0.0006 | 0.0012 | 0.0923 | 0.0460 | 0.0339 | 0.0811 | 0.0003 | 0.0521 | 0.0460 | 0.0116 | 0.0056 | 0.0090 |
| 0.4914 | 0.0000 | 0.0025 | 0.0000 | 0.0001 | 0.0000 | 0.0925 | 0.0039 | 0.0295 | 0.0017 | 0.0000 | 0.0393 | 0.0039 | 0.0241 | 0.0074 | 0.0038 |
| 0.3049 | 0.0000 | 0.0041 | 0.0000 | 0.0000 | 0.0000 | 0.0568 | 0.0065 | 0.0379 | 0.0069 | 0.0000 | 0.0242 | 0.0065 | 0.0192 | 0.0051 | 0.0057 |
| 0.1456 | 0.0001 | 0.0989 | 0.0001 | 0.0006 | 0.0013 | 0.0697 | 0.0234 | 0.0134 | 0.0926 | 0.0001 | 0.0225 | 0.0234 | 0.0035 | 0.0014 | 0.0155 |
| 0.3896 | 0.0000 | 0.0331 | 0.0001 | 0.0028 | 0.0004 | 0.0809 | 0.0151 | 0.0421 | 0.0056 | 0.0001 | 0.0494 | 0.0151 | 0.0132 | 0.0064 | 0.0102 |
| 0.2420 | 0.0000 | 0.0471 | 0.0001 | 0.0008 | 0.0006 | 0.0475 | 0.0984 | 0.0680 | 0.0343 | 0.0001 | 0.0216 | 0.0984 | 0.0046 | 0.0023 | 0.0229 |
| 0.1604 | 0.0000 | 0.0170 | 0.0001 | 0.0000 | 0.0000 | 0.0458 | 0.0331 | 0.0187 | 0.0029 | 0.0001 | 0.0140 | 0.0331 | 0.0052 | 0.0008 | 0.0090 |
| 0.2080 | 0.0000 | 0.0165 | 0.0001 | 0.0001 | 0.0000 | 0.0618 | 0.0131 | 0.0217 | 0.0204 | 0.0001 | 0.0183 | 0.0131 | 0.0086 | 0.0030 | 0.0087 |
| 0.2831 | 0.0000 | 0.0368 | 0.0003 | 0.0003 | 0.0002 | 0.0764 | 0.0289 | 0.0900 | 0.0454 | 0.0003 | 0.0286 | 0.0289 | 0.0080 | 0.0038 | 0.0148 |
| 0.3632 | 0.0000 | 0.0056 | 0.0001 | 0.0001 | 0.0000 | 0.0934 | 0.0031 | 0.0261 | 0.0427 | 0.0001 | 0.0412 | 0.0031 | 0.0199 | 0.0070 | 0.0028 |
| 0.2830 | 0.0000 | 0.0438 | 0.0001 | 0.0000 | 0.0000 | 0.0862 | 0.0109 | 0.0398 | 0.0243 | 0.0001 | 0.0303 | 0.0109 | 0.0084 | 0.0035 | 0.0081 |
| 0.2376 | 0.0000 | 0.0430 | 0.0001 | 0.0011 | 0.0006 | 0.0602 | 0.0809 | 0.0728 | 0.0374 | 0.0001 | 0.0232 | 0.0809 | 0.0052 | 0.0020 | 0.0202 |
| 0.4878 | 0.0000 | 0.0128 | 0.0003 | 0.0042 | 0.0006 | 0.1059 | 0.0131 | 0.0436 | 0.0423 | 0.0003 | 0.0662 | 0.0131 | 0.0234 | 0.0124 | 0.0045 |
| 0.2664 | 0.0000 | 0.0730 | 0.0007 | 0.0007 | 0.0012 | 0.0816 | 0.0508 | 0.0383 | 0.0282 | 0.0007 | 0.0321 | 0.0507 | 0.0086 | 0.0063 | 0.0132 |
| 0.2765 | 0.0000 | 0.0663 | 0.0003 | 0.0001 | 0.0004 | 0.0519 | 0.0518 | 0.0212 | 0.0212 | 0.0003 | 0.0307 | 0.0518 | 0.0035 | 0.0034 | 0.0178 |
| 0.2796 | 0.0000 | 0.0694 | 0.0003 | 0.0014 | 0.0002 | 0.0688 | 0.0100 | 0.0463 | 0.0234 | 0.0003 | 0.0346 | 0.0100 | 0.0122 | 0.0041 | 0.0032 |
| 0.3988 | 0.0000 | 0.0076 | 0.0002 | 0.0210 | 0.0005 | 0.0943 | 0.1023 | 0.0437 | 0.0060 | 0.0002 | 0.0347 | 0.1023 | 0.0471 | 0.0073 | 0.0045 |

**Supplementary Table 4: Key Bacterial Feature Relative Abundance for Female Mice.** Heading identifies lowest taxonomic level. Table includes all designated phyla and features with a significant model.
